# Detecting *ALK* , *ROS1*, and *RET* fusions and the *METΔex14* splicing variant in liquid biopsies of non‐small‐cell lung cancer patients using RNA‐based techniques

**DOI:** 10.1002/1878-0261.13468

**Published:** 2023-06-06

**Authors:** Ana Giménez‐Capitán, Estela Sánchez‐Herrero, Lucía Robado de Lope, Andrés Aguilar‐Hernández, Ivana Sullivan, Virginia Calvo, Irene Moya‐Horno, Santiago Viteri, Carlos Cabrera, Cristina Aguado, Noelia Armiger, Joselyn Valarezo, Clara Mayo‐de‐las‐Casas, Noemí Reguart, Rafael Rosell, Mariano Provencio, Atocha Romero, Miguel A. Molina‐Vila

**Affiliations:** ^1^ Pangaea Oncology, Laboratory of Oncology Dexeus University Hospital Barcelona Spain; ^2^ Atrys Health Barcelona Spain; ^3^ Liquid Biopsy Laboratory Biomedical Sciences Research Institute Puerta de Hierro‐Majadahonda Madrid Spain; ^4^ Dr Rosell Oncology Institute Quirón Dexeus University Hospital Barcelona Spain; ^5^ Hospital de la Santa Creu i Sant Pau Barcelona Spain; ^6^ Medical Oncology Department Hospital Universitario Puerta de Hierro‐Majadahonda Spain; ^7^ Hospital Universitario General de Cataluña Grupo Quirón Sant Cugat del Vallés Spain; ^8^ UOMI Cancer Center Clínica Mi Tres Torres Barcelona Spain; ^9^ Hospital Clínic de Barcelona Spain; ^10^ Hospital Germans Trias i Pujol Health Sciences Institute and Hospital (IGTP) Barcelona Spain

**Keywords:** digital PCR, gene fusions, liquid biopsy, lung cancer, nCounter assay, splicing variants

## Abstract

*ALK*, *ROS1*, and *RET* fusions and *MET*∆ex14 variant associate with response to targeted therapies in non‐small‐cell lung cancer (NSCLC). Technologies for fusion testing in tissue must be adapted to liquid biopsies, which are often the only material available. In this study, circulating‐free RNA (cfRNA) and extracellular vesicle RNA (EV‐RNA) were purified from liquid biopsies. Fusion and *MET*∆ex14 transcripts were analyzed by nCounter (Nanostring) and digital PCR (dPCR) using the QuantStudio^®^ System (Applied Biosystems). We found that nCounter detected *ALK*, *ROS1*, *RET*, or *MET*∆ex14 aberrant transcripts in 28/40 cfRNA samples from positive patients and 0/16 of control individuals (70% sensitivity). Regarding dPCR, aberrant transcripts were detected in the cfRNA of 25/40 positive patients. Concordance between the two techniques was 58%. Inferior results were obtained when analyzing EV‐RNA, where nCounter often failed due to a low amount of input RNA. Finally, results of dPCR testing in serial liquid biopsies of five patients correlated with response to targeted therapy. We conclude that nCounter can be used for multiplex detection of fusion and *MET*∆ex14 transcripts in liquid biopsies, showing a performance comparable with next‐generation sequencing platforms. dPCR could be employed for disease follow‐up in patients with a known alteration. cfRNA should be preferred over EV‐RNA for these analyses.

## Graphical abstract

Next‐generation sequencing (NGS) is employed to detect clinically relevant fusions and splicing variants in cancer patients but shows insufficient sensitivity in liquid biopsies. Our study reveals that nCounter can efficiently detect these alterations in liquid biopsies and shows a strong correlation with tissue genotyping. We also demonstrate that digital PCR can be used for disease monitoring in fusion‐specific patients.

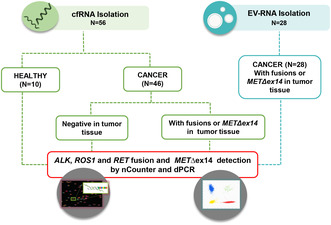

**Abbreviations**
ASC: ascites
cDNA: complementary DNA
cfDNA: circulating‐free DNA
cfRNA: circulating‐free RNA
CSF: cerebrospinal fluid
dPCR: digital PCR
EV‐RNA: extracellular vesicle RNA
EVs: extracellular vesicles
FFPE: formalin‐fixed paraffin‐embedded
FISH: fluorescence *in situ* hybridization
HK: housekeeping
IHC: immunohistochemistry
LOD: limit of detection
MAF: mutant allele frequency
NGS: next‐generation sequencing
NSCLC: non‐small‐cell lung cancer
PCR: polymerase chain reaction
PE: pleural effusion
PFS: progression‐free survival
TKI: tyrosine kinase inhibitor
WT: wild‐type

## 1 Introduction

Lung cancer is one of the most common malignancies and the first cause of tumor‐related deaths, representing almost 25% of them [1]. Despite the advances in imaging techniques, most cases are diagnosed in advanced stages and have a dismal prognosis, with a 7% survival rate at 5 years [2]. More than 80% of lung tumors are histologically adenocarcinomas, squamous cell carcinomas, or large cell carcinomas, which are grouped as non‐small‐cell lung cancers (NSCLCs). Several genetic alterations have been demonstrated to be oncogenic drivers in NSCLC, including point mutations, deletions, insertions, or gene fusions. Specifically, in Western countries, 45% of NSCLC tumors harbor mutations in KRAS proto‐oncogene, GTPase (*KRAS*), epidermal growth factor receptor (*EGFR*) or B‐Raf proto‐oncogene, serine/threonine kinase (*BRAF*) genes; while driver gene fusions and splicing variants are present in 10–15% of patients. The most common gene fusions in NSCLC involve three genes codifying membrane receptors, namely anaplastic lymphoma receptor tyrosine kinase (*ALK*) (5–7% frequency), ROS proto‐oncogene 1, receptor tyrosine kinase (*ROS1*) and ret proto‐oncogene (*RET*) (1–2% each); while neurotrophic receptor tyrosine kinase 1/2/3 (*NTRK1/2/3*) fusions are rare (< 0.1%). Regarding splicing variants, *MET* exon 14 skipping (*MET*Δex14) appears in 3–4% of NSCLC patients. These oncogenic fusions, together with the *MET*Δex14, are mutually exclusive with other drivers and codify constitutively active kinases that represent actionable alterations for FDA‐approved therapies [3, 4, 5, 6, 7, 8, 9, 10, 11].

The identification and characterization of gene fusion and splicing variants in clinical biopsies were initially performed using methodologies targeting single alterations. These techniques, based on the polymerase chain reaction (PCR), are still in use in many laboratories and include digital PCR (dPCR), which stands out for its high sensitivity. However, the ever‐growing list of targetable fusions and variants encouraged the development of multiplex techniques to interrogate more than one alteration in each assay, such as RNA and DNA‐based next‐generation sequencing (NGS) or the RNA‐based nCounter platform, which has been successfully applied for the detection of fusion rearrangements and splicing variants in formalin‐fixed paraffin‐embedded (FFPE) tumor tissue samples [12, 13, 14].

Unfortunately, the availability of tumor tissue for genetic analyses is compromised in a significant number of advanced‐stage NSCLC patients, which cannot be biopsied or only have cytological specimens or small biopsies available. In addition, repeated biopsies to monitor the course of the disease or to detect the emergence of resistance are often impossible to obtain. In this setting, liquid biopsies represent a minimally invasive, safe, and sensitive alternative that is increasingly being used for the study of tumor‐associated molecular alterations. Liquid biopsy samples include several biological materials such as blood, pleural effusion (PE), ascites (ASC), or cerebrospinal fluid (CSF) and different components within these fluids, including circulating‐free nucleic acids, circulating tumor cells, extracellular vesicles (EVs), or platelets. All these materials and components can be used for genetic testing, although circulating‐free DNA and RNA (cfDNA and cfRNA) purified from plasma [15, 16] or RNA from platelets [17] are most commonly used. However, the detection of gene fusions and splicing variants in liquid biopsies is not yet routine clinical practice in many hospitals and the performance of NGS platforms is typically inferior compared with FFPE tissue biopsies [17, 18, 19, 20], highlighting the need for new methodological approaches that can be successfully applied in this setting.

In this study, we have tested nCounter and dPCR in liquid biopsies from advanced NSCLC patients. Our results demonstrate that these techniques can be employed for the detection of *ALK*, *ROS1*, and *RET* fusions and *MET*Δex14 splicing variant in fluids.

## 2 Materials and methods

### 2.1 Study cohort, clinical samples, and cell lines

Fifty‐six liquid biopsies of healthy donors and patients with NSCLC, breast, prostate, and pancreatic cancer were used for cfRNA purification, and plasma samples from 28 additional NSCLC patients were selected for EV‐RNA extraction (Tables 1 and 2). All samples were collected during routine clinical practice from May 2016 to February 2022. The 56 liquid biopsies used for cfRNA were obtained from five hospitals in Barcelona, Spain; while the 28 plasma samples for EV‐RNA purification were collected in Hospital Puerta del Hierro in Madrid, Spain (Table S1). The study, conducted in accordance with the principles of the Declaration of Helsinki, was approved by the Ethics Committees of Hospital Puerta de Hierro (internal code PI 118/22) and Hospital Universitario Dexeus (approval number 51/2018). All patients provided their appropriate written informed consent.

**Table 1:** Characteristics of the patients with circulating‐free (cfRNA) samples included in the study.

Characteristics	Tissue‐positive patients		Negative controls	
	*N* = 40	%	*N* = 16	%
Age/years				
Median age	58		37	
Range	33–89		22–78	
Gender				
Male	13	32.5	5	31.3
Female	27	67.5	11	68.7
Smoking status				
Smoker	2	5.0	5	31.3
Former	10	25.0	2	12.5
Never	4	10.0	6	37.5
Unknown	24	60.0	3	18.7
Type of tumor				
NSCLC	40	100	3	18.75
Breast cancer	0		1	6.25
Prostate cancer	0		1	6.25
Pancreatic cancer	0		1	6.25
No tumor	0		10	62.5
Stage				
I–IIIA	0		1	6.25
IIIB–IV	39	97.5	4	25.0
Unknown	1	2.5	1	6.25
No tumor	0		10	62.5
Type of fluid				
Plasma	32	80.0	12	75.0
Pleural effusion (PE)	5	12.5	3	18.75
Cerebrospinal fluid (CSF)	3	7.5	0	
Ascites (ASC)	0		1	6.25
Alterations in FFPE tissue				
*ALK*	14	35.0	0	
*ROS1*	9	22.5	0	
*RET*	12	30.0	0	
*MET*Δex14	5	12.5	0	
*No fusions or splicing variant*	0		16	100

**Table 2:** Characteristics of the patients with extracellular vesicle RNA (EV‐RNA) samples evaluated in the study.

Characteristics	*N* = 28	%
Type of tumor		
NSCLC	26	92.85
Stage		
IIIB–IV	28	100.0
Age/years		
Median age	56.5	
Range	36–86	
Gender		
Male	11	39.28
Female	17	60.71
Smoking status		
Smoker	5	17.85
Former	5	17.85
Never	18	64.28
Type of fluid		
Plasma	28	100.0
Alterations in FFPE tissue in FFPE tissue		
*ALK*	26	92.86
*RET*	1	3.58
*MET*Δex14	1	3.58

Eight cell lines were used as reference standards for validation purposes (Table S2). All cell lines were purchased from the ATCC with the exception of PC9, which was provided by F. Hoffman‐La Roche Ltd (Basel, Switzerland) with the authorization of Dr. Mayumi Ono (Kyushu University, Fukuoka, Japan). Cells were cultured in RPMI medium with 10% fetal bovine serum under standard conditions. Cell lines were routinely tested for mycoplasm contamination and only mycoplasm‐free cultures were used in the study. Cell lines were authenticated by analyzing 20 polymorphisms on DNA using a 30‐gene NGS panel [21] and by nCounter on RNA using the custom panel described below. In all cases, the genotypes of the cells were coincident.

### 2.2 RNA isolation from clinical samples and cell lines

For cfRNA isolation, peripheral whole‐blood samples were collected in 10‐mL sterile BD Vacutainer^®^ K2E tubes (BD, Plymouth, UK), containing 18 mg of EDTA as anticoagulant. PE, CSF, and ASC samples were collected in 10‐mL plastic sterile tubes with no additives or anticoagulants. Collection tubes containing the liquid biopsy samples (blood, PE, CSF, or ASC) were centrifuged for 10 min at 500 ***g*** within 24 h. Then, using a Pasteur pipette, the supernatant was recovered, transferred to a new sterile tube, and recentrifuged using the same conditions. The cfRNA was immediately isolated from 4 mL of the supernatant of the second centrifugation using the QIAsymphony^®^ DSP Virus/Pathogen Midi Kit in a QIAsymphony robot (Qiagen, Hilden, Germany), following the manufacturer's instructions. The maximum 24‐h interval between blood collection and RNA purification was decided based on a stability study performed in‐house (Table S3), which was in line with previous publications about RNA stability in K2‐EDTA tubes [22, 23, 24, 25]. The automatic extraction procedure is based on magnetic particles and involves bind, wash, and elution steps. Final elution volume was 50 μL in all cases. RNA concentration was estimated by Qubit™, and 5 μL was retrotranscribed to cDNA. We used 2.5 ng·μL^−1^ of cfRNA as the maximum input for the retrotranscription (RT) reaction and diluted the starting RNA to 2.5 ng·μL^−1^ if it had a higher concentration. The RT reaction was performed with the nCounter^®^ Low RNA Input Amplification Kit (NanoString Technologies, Seattle, WA), which contains commercial retrotranscription primer and enzyme mixes. All RNA samples were retrotranscribed and subsequently submitted to nCounter analysis, independently of the quantity or quality of the purified RNA.

EV‐RNA was purified from 28 plasma samples of additional *ALK* (*n* = 26), *RET*, or *MET*Δex14‐positive NSCLC patients. Blood samples were collected in a 10‐mL Streck tube, cells were removed by centrifugation at 2000 ***g*** for 10 min at 4 °C and the supernatant was diluted in PBS (1 : 3) and centrifuged at 110 000 ***g*** for 2 h at 4 °C with no brake to collect EVs. The characterization of plasma‐derived EVs (*N* = 2) was performed by ExoView R100 (NanoView Biosciences) using human tetraspanins kits (EV‐DTETRA‐C). The expression of CD9, CD81, and CD63 EV markers, as well as *ALK* fusion protein, was visualized by ExoView Analyzer (NanoView Biosciences, Boston, MA) as published elsewhere [26]. RNA from EVs preparations (50 μL each) was isolated manually after EVs lysis with QIAzol reagent (QIAgen), using exoRNeasy^®^ Serum/Plasma Maxi Kit (QIAgen), following the manufacturer's instructions. RNA was eluted in 14 μL of RNase‐free water and 6.5 μL reverse‐transcribed into complementary DNA (cDNA) using the PrimeScript RT Reagent Kit (TaKaRa, Shiga, Japan), according to the manufacturer's instructions.

Total RNA from cell lines was extracted manually with a high‐purity RNA isolation kit (Roche Diagnostics, Mannheim, Germany) according to the manufacturer's instructions. RNA concentration was estimated using Qubit™. EV‐RNA from the culture media of cell lines was purified as described [27].

Finally, the quality of cfRNA and EV‐RNA samples was evaluated using Agilent RNA 6000 Pico Kit on a 2100 Bioanalyzer (Agilent, Santa Clara, CA, USA; Fig. S1).

### 2.3 nCounter analysis

The nCounter assays for the detection of altered transcripts in FFPE biopsies of cancer patients are based on direct hybridization, without any amplification step [14, 28, 29]. However, the number of aberrant transcripts in liquid biopsy samples is expected to be low and highly diluted in wild‐type DNA. Consequently, we decided to add a 30‐cycle preamplification step to our workflow. The cDNAs retrotranscribed from cfRNA and EV‐RNAs were preamplified using the corresponding enzyme mix of the nCounter^®^ Low RNA Input Amplification Kit (NanoString Technologies) and a custom primer pool targeting housekeeping (HK), fusion and splicing transcripts (Table S4). Finally, preamplified cDNA was denatured at 95 °C for 5 min and hybridized at 67 °C for 18–24 h with a mixture of commercial biotinylated capture tags (NanoString Technologies) and a custom nCounter panel of fluorescently labeled reporter probes, following the Elements Chemistry [14, 29]. The preamplification, denaturation, and hybridization steps were performed in a Verity thermal cycler (Applied Biosystems, South San Francisco, CA, USA). The custom nCounter panel contained probes for 3 HK genes [glyceraldehyde‐3‐phosphate dehydrogenase (*GAPDH*), Mitochondrial Ribosomal Protein L19 (*MRPL19*) and proteasome 26S subunit, ATPase 4 (*PSMC4*)], 23 pairs of probes specific for the most common fusion transcripts (7 *ALK*, *6 RET*, and 10 *ROS1*), probes for *MET‐*[wild‐type (wt)] and *MET*Δex14 transcripts and six negative control probes for background correction, as described (Table S5) [12, 14]. Finally, capture, clean up, and digital data acquisition of the hybridized mixture were performed in the nCounter Prep Station™ and Digital Analyzer™ (NanoString Technologies). Raw nCounter counts were exported to Excel 2016 (Microsoft, Redmond, WA) using nsolver software v4.0 (NanoString Technologies).

Samples were considered evaluable if the geometric mean of HK counts was higher than 30. A sample was considered positive for a specific fusion if the counts of the corresponding transcript were higher than the arithmetic mean plus six times the standard deviation (6xSD) of the counts in the negative samples. For *MET*Δex14 splicing variant, the cut‐off for positivity was established as a 1.0 ratio of *MET*Δex14 vs. wild‐type *MET* counts [12]. To determine the technical variation of the nCounter technique, six aliquots of a *RET*‐positive cfRNA were analyzed in different runs and the results plotted as a Levey‐Jennings chart (Fig. S2).

### 2.4 Digital PCR

dPCR reactions for fusion transcript detection were performed in a 15‐μL volume containing 7.5 μL of 2× QuantStudio 3D Master Mix, 0.5 μL of 40× predesigned TaqMan^®^ assay for endogenous pumilio RNA binding family member 1 (*PUM1*) gene, 0.5 μL of 40X commercially available predesigned or custom TaqMan^®^ assays and 6.5 μL of cDNA. dPCR reaction for *MET*∆ex14 includes 0.5 μL of 40× custom TaqMan^®^ assay for *MET*∆ex14 instead of *PUM1*. TaqMan^®^ assays used for specific genomic alterations are listed in Table S6. The limit of detection for specific assays has been published elsewhere [26]. Then, 14.5 μL of the PCR mixture was loaded onto QuantStudio 3D Digital PCR 20 K chips by QuantStudio™ 3D dPCR Chip Loader (Applied Biosystems). For each run, a blank (containing no cDNA), a negative control cDNA wt, and a positive control (if available) were included. PCR reactions were performed according to the following thermal cycler conditions: 10 min at 96 °C, then 45 cycles of 2 min at 56 °C, and 30 s at 98 °C. Finally, samples were elongated for 10 min at 72 °C and kept at 22 °C for at least 30 min. Chips were read in two different QuantStudio™ 3D dPCR Instruments. Final data were visualized and analyzed by QuantStudio^®^ 3D Analysis Suite™ Cloud Software, adjusting call assignments for each cluster manually, when needed. Results were reported as the mutant allele frequency (MAF), defined as the number of mutant cDNA molecules relative to the sum of total cDNA molecules (mutant and wt). To determine the technical variation of the dPCR technique, 10 replicates of a control sample (H2228‐derived RNA) were analyzed and the results plotted as a Levey‐Jennings chart (Fig. S2).

### 2.5 Statistical analysis

The agreement between tissue genotyping and nCounter was assessed using the kappa coefficient values and the corresponding 95% confidence intervals (95% CI). Differences in median total counts between samples from fusion or *MET*∆ex14‐positive patients and ‐negative controls were evaluated using the Mann–Whitney *U* test and the frequencies in the detection of alterations by nCounter vs. dPCR by two‐tailed *Z* score. graphpad prism v6.0 (GraphPad Software Inc., San Diego, CA) was used for all statistical analyses; *P*‐values of < 0.05 were considered statistically significant.

## 3 Results

### 3.1 Limit of detection (LOD)

In order to determine the limit of detection of nCounter for fusions and *MET*∆ex14, we used a mixture of total RNAs from five positive cell lines spiked 1 : 1 into a pan‐negative plasma cfRNA. The mixture was serially diluted into the same pan‐negative cfRNA. We found that the minimum amount of RNA needed for the detection of *ALK*, *ROS1*, *RET* fusion transcripts, and *MET*Δex14 ranged between 1 and 10 pg·μL^−1^, corresponding to 0.1–0.01% fraction of tumor RNA (Table S7).

The LOD of nCounter was also determined for EV‐RNA. First, we tested the EV‐RNAs purified from the culture media of seven human tumor cell lines. Five of the EV‐RNAs tested positive, corresponding to the media of cells harboring *ALK*, *ROS1*, and *RET* rearrangements and the *MET*∆ex14 splicing variant. By contrast, the two EV‐RNAs from fusion‐negative cells were pan‐negative (Table S8). Then, we spiked EV‐RNA from the *ROS1*‐positive HCC78 into EV‐RNA from the PC9 cell media (*EGFR*‐mutant, wild‐type for fusions and splicing variants). We found that at least 5 pg of EV‐RNA were needed to detect the *ROS1* fusion (Table S9).

### 3.2 Detection of fusion and *MET* ∆ex14 transcripts in cfRNA from clinical samples

**Fig. 1:**
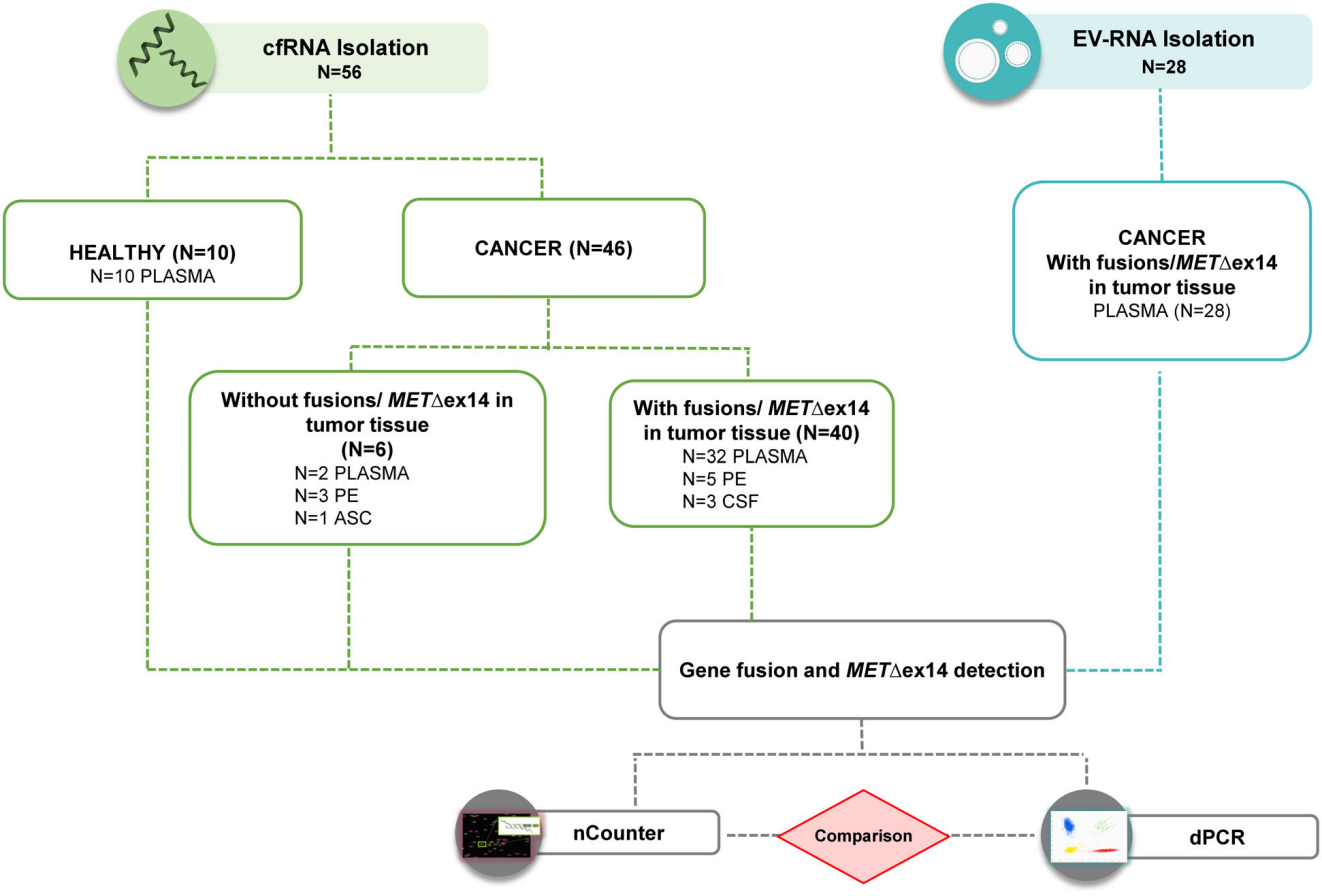
Flow chart of the patient cohorts for the cfRNA (*n* = 56) and EV‐RNA (*n* = 28) studies. In the cfRNA study, a total of 56 liquid biopsies were included, 10 from healthy individuals and 46 from cancer patients. Of the 46 samples from patients, 40 samples were positive for fusions or *MET*∆ex14 in tumor tissue, while the remaining six were negative. In the case of EV‐RNA study, plasma samples from 28 additional patients with fusions or *MET*∆ex14 in tissue were included. All samples in both studies were analyzed by nCounter and digital PCR. ASC, ascites; cfRNA, cell‐free RNA; CSF, cerebrospinal fluid; dPCR, digital PCR; EV‐RNA, extracellular vesicle RNA; PE, pleural effusion.

A total of 56 liquid biopsy samples were used for cfRNA purification and validation of the nCounter methodology (Fig. 1). Of them, 40 samples were collected from fusion or *MET*∆ex14‐positive patients previously genotyped in FFPE tissue by nCounter, FISH, IHC, and/or NGS (Table S10). Most of them corresponded to plasma (*n* = 32), but PE (*n* = 5) and CSF (*n* = 3) were also represented. The remaining 16 liquid biopsies were used as controls: 10 samples from healthy donors and six samples collected from lung (*n* = 3), breast, prostate, and pancreatic cancer patients pan‐negative for fusions and *MET*∆ex14 in tumor tissue (Table 1).

The 56 liquid biopsies included in the study yielded valid results by nCounter, presenting HK levels above the threshold previously established (see Section 2). The concentration in the cfRNA extracts, as determined by Qubit, ranged from 0.4 to 70 ng RNA·μL,^−1^ and samples above 2.5 ng·μL^−1^ were diluted before analysis to reach this concentration. No correlation was found between the concentration of cfRNA extracts or the total amount of input RNA and the HK levels (Table S11 and Fig. S3A). Regarding quality, 10 cfRNA samples were submitted to bionanalyzer, results are presented in Fig. S1A,B and Table S11.

**Fig. 2:**
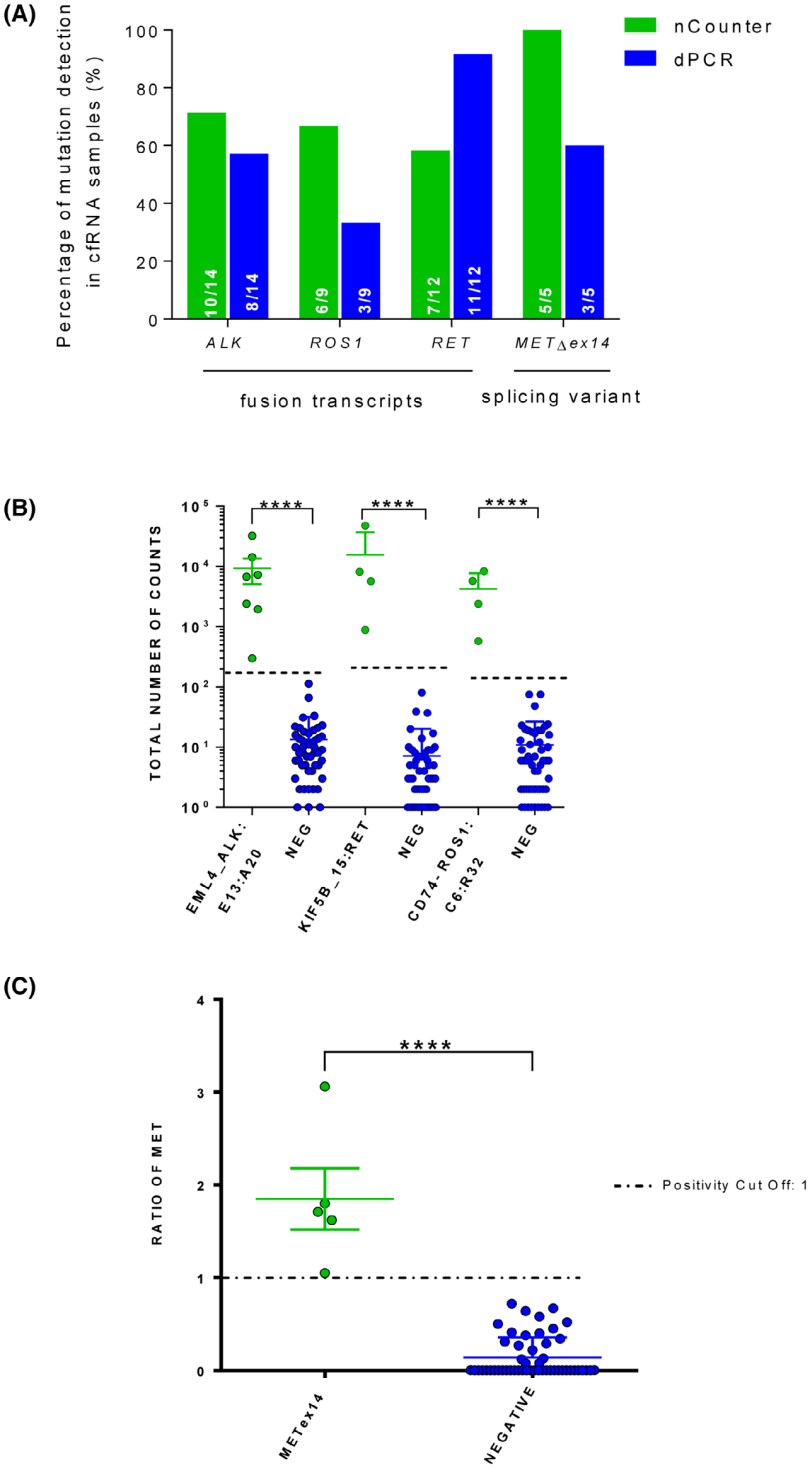
Results of the nCounter and dPCR analyses of cfRNA samples. (A) Percentage of fusion and *MET*Δex14 detection in cfRNA samples by nCounter and dPCR. (B) Comparison of the total number of counts by nCounter of positive vs. negative samples for *ALKv1*, *CD74‐ROS1*, and *KIF5B‐RET* rearrangements (****ρ < 0.0001 in a two‐tailed Mann–Whitney *U* test). Error bars indicated mean ± SEM while dotted lines indicate the threshold. (C) Comparison of the ratio of *MET*∆ex14 variant counts by nCounter in positive vs. negative patients (****ρ < 0.0001 in a two‐tailed Mann–Whitney *U* test). Error bars indicated mean ± SEM while dotted lines indicate the positivity threshold. cfRNA, cell‐free RNA; dPCR, digital PCR.

All 16 control samples were negative for the 23 probes included in the nCounter panel. Among the 40 cfRNA samples from positive cancer patients, we could identify altered transcripts in 28; including 10 *ALK*, 6 *ROS1*, and 7 *RET* fusion transcripts and 5 *MET*∆ex14 splicing variants (Fig. 2A). As expected, positivity for aberrant transcripts was mutually exclusive and the alterations found in cfRNA were always coincident with those previously found in FFPE biopsies. The most common *ALK* variant was EMAP‐like 4 (*EML4*) *EML4‐ALK v*1 (*E13:A20*) (*n* = 7), followed by *v*3 (*E6a/b:A20*) (*n* = 2) and *v*5 (*E18:A20*) (*n* = 1); whereas the genes involved in *ROS1* fusions were CD74 molecule (*CD74*) (*n* = 4), solute carrier family 34 member 2 (*SCL34A2*) and stearoyl‐CoA desaturase 4 (*SCD4*) (*n* = 1 each). In *RET*‐positive samples, kinesin family member 5B (*KIF5B*) (*n* = 4) and coiled‐coil domain containing 6 (*CCDC6*) (*n* = 3) were the partners identified. The detection of altered transcripts by nCounter in liquid biopsy showed a sensitivity vs. tissue ranging from 58% for *RET* fusions to 100% for *MET*∆ex14, with specificities of 100% in all cases and Cohen's kappa from 0.69 to 1 (Table 3). Overall, nCounter demonstrated a 70% (CI = 54.6–81.9) sensitivity and 100% (CI = 97.8–100) specificity, with an almost perfect agreement with genotyping in tissue (Cohen's Kappa = 0.8; 95%CI = 0.66–0.92).

**Table 3:** Concordance of *ALK*, *ROS1*, *RET*, and *MET*∆ex14 detection in circulating‐free RNA (cfRNA) liquid biopsy vs. tissue by nCounter in absolute number of samples.

Genes	*ALK*	*ROS1*	*RET*	*MET*Δex14	Overall
No. concordant samples	52	53	51	56	212
No. discordant samples	4	3	5	0	12
Diagnostic sensitivity	71.4% (CI = 45.3–88.3)	67.6% (CI = 35.8–87.9)	58.3% (CI = 30.4–86.2)	100% (CI = 56.6–100)	70% (CI = 54.6–81.9)
Diagnostic specificity	100% (CI = 91.6–100)	100% (CI = 92.4–100)	100% (CI = 91.9–100)	100% (CI = 92.6–100)	100% (CI = 97.8–100)
Concordance	92.85%	94.64%	91.07%	100%	94.41%
Cohen's κ	0.79 (CI = 0.53–1.04)	0.77 (CI = 0.51–1.03)	0.69 (CI = 0.44–0.94)	1 (CI = 0.74–1.27)	0.8 (CI = 0.66–0.92)

The median total counts of liquid biopsy samples positive for *ALKv1*, *CD74‐ROS1*, and *KIF5B‐RET* fusion transcripts were 6756, 4063, and 6911; compared with 8, 6, and 3 counts for negative samples, respectively. Differences were statistically significant in all cases (*P* < 0.001 by Mann–Whitney *U* test, Fig. 2B). Similar results were obtained for the rest of the *ALK*, *ROS1*, and *RET* variants. Regarding *MET*∆ex14, the median ratio of the skipping vs. the wt probe was 0.1 in control samples compared with 1.7 in positive samples, the difference was again significant (*P* < 0.001 by Mann–Whitney *U* test, Fig. 2C). Among liquid biopsies, positive PE samples generally showed higher counts by nCounter than blood samples, although the trend did not reach statistical significance, probably due to the small number of specimens (Fig. S3B).

Next, we investigated whether the detection of aberrant transcripts in blood could be dependent on the quantity of cfRNA isolated. To this end, we compared the geometric mean of HK gene counts in the cfRNAs from patients with fusions or *MET*∆ex14 in tissue, but we did not find significant differences between plasma samples positive and negative by nCounter (Fig. S4).

**Fig. 3:**
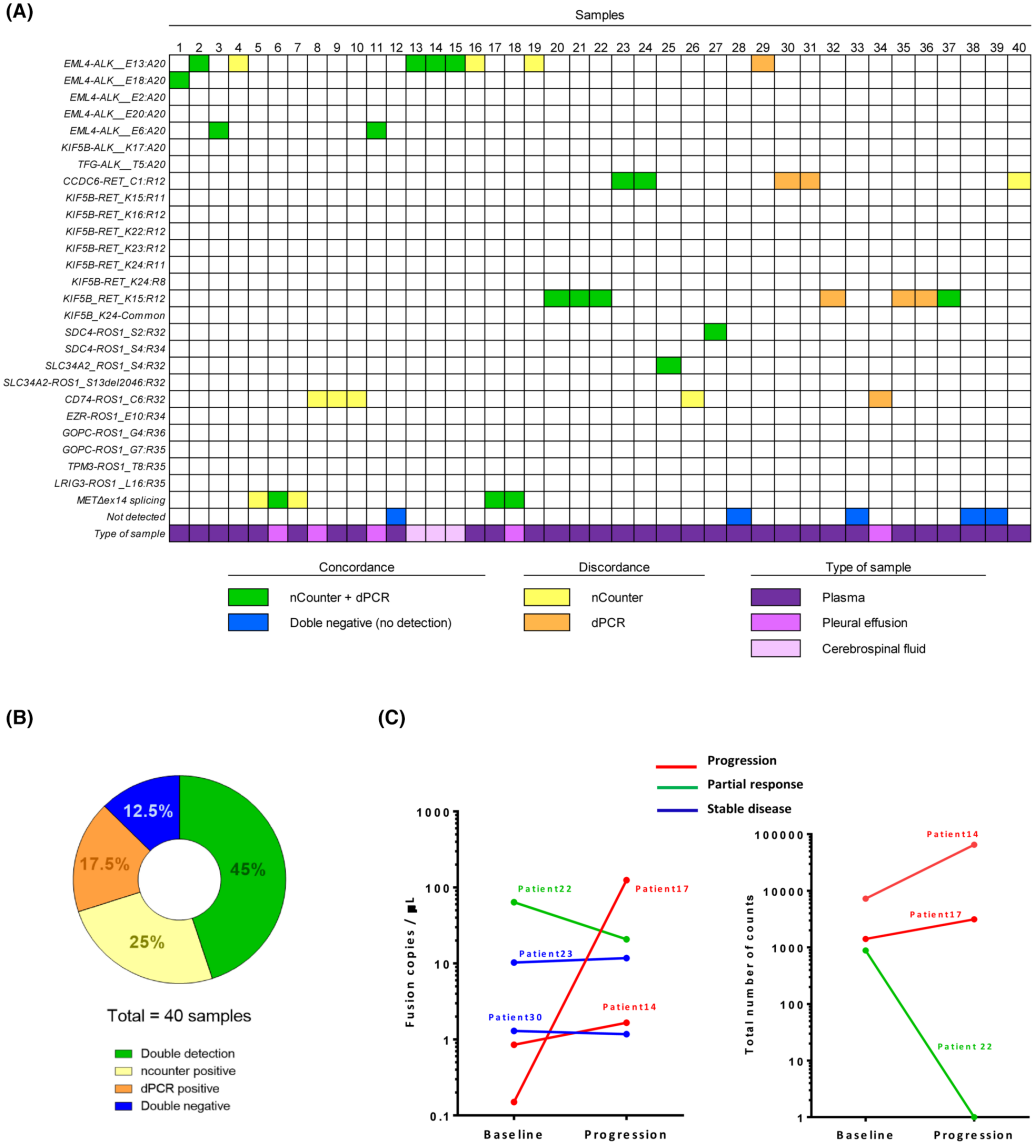
Concordance of nCounter and dPCR results in cfRNA and EV‐RNA samples. (A) Heatmap of the liquid biopsies from tumor‐positive patients included in the orthogonal study (*n* = 40). (B) Summary of the results presented in (A). (C) Results of the fusion analysis of serial liquid biopsy samples by dPCR (left panel) and nCounter (right panel). cfRNA, cell‐free RNA; dPCR, digital PCR; EV‐RNA, extracellular vesicle RNA.

Finally, we used dPCR as an orthogonal method to analyze the 40 cfRNA samples from tissue‐positive patients. Aberrant transcripts were identified in 25 of them (62.5%) and the percentage of detection of genetic alterations by dPCR was slightly lower compared with nCounter, except for *RET* fusion transcripts (Fig. 3A). The overall concordance of the two techniques was 57.5% (23/40), being 71.1% (10/14) if only *ALK* samples were considered, with three cases negative by dPCR but positive by nCounter and one case positive by dPCR and negative by nCounter (Table S12). Furthermore, the combination of nCounter and dPCR techniques enabled the detection of the analyzed fusion transcripts and *MET*Δex14 splicing variant in 87.5% of the samples (Fig. 3B).

### 3.3 Detection of fusion transcripts in EV‐RNA from clinical samples

Extracellular vesicles were isolated from the blood of 28 additional NSCLC patients with tumor biopsies positive for *ALK* rearrangements (*n* = 26), *RET* rearrangements (*n* = 1), or *MET*∆ex14 (*n* = 1) by NGS, FISH, IHC, or qPCR (Table 2). NGS analysis of the FFPE samples was able to identify the fusion variant in 12 cases; the remaining 16 FFPE biopsies did not have enough material for NGS (Table S13). The expression of CD9, CD81, and CD63 tetraspanins was confirmed in EV‐preparations (*n* = 2) as previously reported [26]. EV‐RNA was subsequently purified and analyzed by dPCR and nCounter (Fig. 1). Nine EV‐RNA preparations were also tested by bioanalyzer (Fig. S1C,D).

In the case of dPCR, 4/28 EV‐RNA samples (14.3%) were not evaluable due to low or no detection of endogenous gene (*PUM1*) (< 0.5 copies·μL^−1^). Oncogenic variants were identified in 13/28 samples (46.4%) using primers targeting the aberrant transcript present in the tissue (4/9) or the most common variants when characterization in tissue was not possible (9/15). Specifically, the oncogenic transcripts found in EV‐RNAs were *EML4‐ALKv*3 (*n* = 6), *v*1 (*n* = 4) and *v*2 (*E20:A20*) (*n* = 1), *KIF5B‐RET* (*K15:R12*) (*n* = 1), and *MET*∆ex14 splicing variant (*n* = 1) (Fig. S5A). In the case of nCounter, 10/28 samples were considered not evaluable due to low HK counts (Fig. S5B). Among the 18 samples successfully analyzed, aberrant transcripts were detected in four (22.2%) (Fig. S5C). Particularly, we found alterations in *ALK* (*KIF5B‐ALK_K17:A20* and *EML4‐ALK_E20:A20*), *RET* (*KIF5B‐RET_K15:R12*), and *MET* (*MET∆ex14*) (Fig. S5A).

### 3.4 dPCR and nCounter for patient follow‐up

Blood samples at baseline and evaluation of response to TKIs were available for five patients harboring *ALK* (*n* = 1) or *RET* (*n* = 3) fusions and *MET∆ex14* (*n* = 1), as determined in FFPE tissue. In the two cases with progressive disease, the dPCR mutant copies·μL^−1^ corresponding to the altered transcripts in blood increased. By contrast, dPCR mutant copies·μL^−1^ remained in the same range of magnitude in the two patients that experienced stabilization of the disease while a five‐fold decrease in mutant copies·μL^−1^ was observed in a patient that showed a partial response upon TKI treatment (Fig. 3C). A similar trend was observed in the nCounter counts in three of the patients, while the serial samples were consistently negative in the other two.

## 4 Discussion

Actionable alterations in advanced nonsquamous NSCLC include *ALK*, *ROS1*, and *RET* rearrangements and *MET*∆ex14 splicing variant. Gene fusions involving *ALK* and *ROS1* are predictive biomarkers of response to first‐line tyrosine kinase inhibitors (TKI) including alectinib, lorlatinib, crizotinib, or ceritinib [6, 8, 10] and are routinely tested in advanced NSCLC patients. In addition, U.S. Food and Drug Administration (FDA) and European Medicines Agency (EMA) have approved selpercatinib and praselisib [30] for *RET*‐positive patients and tepotinib and capmatinib for tumors harboring the *MET∆*ex14 splicing variant [31]. Although tumor biopsies should be preferred when testing for these alterations, a significant number of NSCLC patients cannot be biopsied or the tissue available is insufficient for genetic analysis. In this case, liquid biopsies represent a minimally invasive, safe, and easy‐to‐collect alternative. Consequently, there is a need to adapt the technologies usually employed for tissue genotyping to the special requirements of liquid biopsies in order to guarantee acceptable sensitivities and specificities in the clinical setting.

DNA or RNA‐based NGS is currently the multiplex method of choice to determine gene fusions and splicing variants in liquid biopsies, while qPCR‐derived techniques are widely used to detect specific alterations. Our results demonstrate that nCounter and dPCR can also be successfully applied for the detection of fusions and splicing transcripts not only in tissue [12, 14] but also in blood and other fluids. Remarkably, the combination of nCounter and dPCR analysis enabled the detection of aberrant transcripts in 87.5% of the cfRNA samples from patients positive in tumor (Fig. 3B). In particular, nCounter could detect aberrant transcripts in cfRNA at abundances as low as 0.01%, showing 70% sensitivity (CI = 54.6–81.9) and 100% specificity (CI = 97.8–100) vs. tissue. Consequently, nCounter outperforms RT‐PCR and Cobas, which have demonstrated only 21% and 33% sensitivity in cfRNA purified from blood, and is in the range of NGS commercial platforms, which have shown sensitivities around 65% for the detection of *ALK* and *ROS1* fusions in plasma (Table S14). Only the Guardant360 CDx assay has reported in its specification sheet a better sensitivity than nCounter for gene fusions (83%) in liquid biopsies, based on the analysis of a limited number of samples (*n* = 37) [32]. However, several articles describing the performance of Guardant360 CDx in the clinical setting have described lower sensitivities for fusion detection in plasma, ranging from 18% to 44% [33, 34, 35]. Thus, in an extensive study enrolling 323 NSCLC patients, *ALK* fusions were detected only in 5 (1.5%), a frequency significantly lower than the 4–5% reported in tissue biopsies [36]. Contrary to nCounter; Guardant360 CDx, Foundation One Liquid CDx, and other commercially available platforms for fusion detection in blood use cfDNA (Table S14). In addition, nCounter has several advantages over these platforms, including a lower price, a shorter turnaround time (2–3 days), and fewer sample requirements. It can also be performed in‐site, avoiding the need for shipment to a central lab, and used to detect not only fusions [12, 14] but also clinically relevant mutations [37]. However, the nCounter methodology described here also presents some limitations. In contrast to fusion detection in tissue samples, it is exclusively based on a panel of specific probes. No imbalance probes are included, and therefore, fusions with partners unknown or not covered by the panel cannot be detected. This is in contrast with other techniques, such as anchored multiplex PCR, hybrid capture‐based NGS, or the 3/5‐imbalance score of some NGS platforms, which are open systems and can detect fusions with unknown partners [38].

Regarding dPCR, several studies have demonstrated a good agreement between plasma testing using dPCR and tissue genotyping [39, 40]. Moreover, dPCR is also cheaper, simpler, and with a shorter turnaround time than NGS or BEAMing, and can be employed to detect *EGFR* and other clinically relevant mutations. Nevertheless, only a small number of alterations can be simultaneously tested at a time, which constitutes a major limitation [41, 42, 43]. In our study, target variant analysis by dPCR enabled the detection of previously known alterations in 62.5% of cfRNA samples (Fig. 3B). When comparing dPCR with nCounter, we observed a number of discordant blood samples higher than expected (17/40). These 17 discordant cases concentrated in certain types of fusions, particularly *CD74‐ROS1* (*n* = 5), *ALKv1* (*n* = 4), and *RET* (*n* = 6). Remarkably, the five *CD74‐ROS1*‐positive blood samples included in the study were discordant, with nCounter detecting four cases and dPCR only one. By contrast, the two positive samples harboring other *ROS1* variants were concordant. In the case of *ALKv1*, concordance was 50%; with three cases detected exclusively by nCounter and one by dPCR. Again, the three samples positive for other *ALK* variants were detected by both techniques (Fig. 3A). Therefore, it is likely that the design of the commercial assays for dPCR is not fully adequate for fusion detection at very low levels in the case of *CD74‐ROS1* and *ALKv1*. In the case of *RET*, concordance was also 50% but the situation was the opposite, with five samples detected exclusively by dPCR and only one by nCounter. Consequently, the design of the nCounter probes for *CCDC6‐RET* and *KIF5B‐RET* should probably be optimized.

We also quantified by dPCR the aberrant transcripts in serial liquid biopsies of five patients and we observed a correlation with the type of response to targeted therapies. This correlation was also observed in three of the patients using nCounter, while the other two cases were consistently negative by this technique (Fig. 3C). Therefore, dPCR could potentially be useful for monitoring specific druggable alterations in liquid biopsies but does not seem suitable for biomarker screening procedures. However, our results in this respect must be considered as purely exploratory. Additional systematic testing will be required to define clinically relevant thresholds so that ‘true’ changes in MAF at response or progression can be distinguished from differences arising from the inherent technical variability of the assays. A limited number of pilot studies have examined the clinical utility of the longitudinal evaluation of *EML4‐ALK* positivity in blood, with discordant results. In a cohort of 50 French patients, the dynamic change in *ALK*‐positive circulating tumor cells (CTC), as estimated by FISH, did not significantly associate with response to treatment [44]. By contrast, a study in 11 patients revealed a decrease in the MAFs of the *ALK* fusion associated with clinical responses to ensartinib, as determined by DNA‐based NGS. However, in most cases, the fusion did not reappear at progression [45]. Finally, a study using the Guardant 360 platform and enrolling 92 Korean patients found frequent disappearance of the *ALK* positivity in circulating‐free DNA after tyrosine kinase treatment, which was associated with a longer progression‐free survival and overall survival [46].

Finally, our results also indicate that nCounter should preferentially be used in plasma cfRNA rather than in EV‐RNA preparations, which contain less quantity of RNA. All cfRNA samples in our cohort were successfully analyzed by nCounter compared with 35% of nonevaluable EV‐RNAs due to low HK counts. dPCR showed a significantly better performance than nCounter in this type of sample, with 54% sensitivity. Of note, plasma cfRNA is much easier to purify than EV‐RNA, which requires EV‐enrichment step by ultracentrifugation or specially designed kits that might not be available or can be difficult to implement in the clinical setting.

## 5 Conclusions

nCounter can be used for multiplex detection of fusion and *MET∆*ex14 transcripts in liquid biopsies of non‐small‐cell lung cancer patients, and has a performance comparable or superior to next‐generation sequencing platforms in this setting. dPCR, which targets a single alteration, could be employed for disease follow‐up in patients with a known fusion or splicing variant. Finally, cfRNA should be preferred for fusion testing in blood over EV‐RNA, which is present in limited quantities and has a tedious isolation procedure.

## Conflict of interest

AG‐C received some of the reagents used in the project free‐of‐charge from NanoString Technologies. VC has received research payment or honoraria for lectures, presentations, speaker's bureaus, manuscript writing, or educational events from Roche, BMS, MSD, Astrazeneca, Takeda, Pfizer, Lilly, and Boehringer Ingelheim. NR has received fees for consultancy/advisory roles from MSD, BMS and Pfizer, and research funding from Pfizer and Novartis. MP has received research funding from BristolMyers Squibb, Roche, Boehringer Ingelheim, and Pierre Fabre (Inst); consulting fees from Astrazeneca, Takeda, Bristol Myers Squibb, MSD; and support for attending meetings from Bristol Myers Squibb. AR has received research funding from BristolMyers Squibb Foundation, Boehringer Ingelheim, and Takeda; consulting fees from Astrazeneca and Takeda; and support for attending meetings from Thermofisher Scientific and Bristol Myers Squibb. MAM‐V has received research funding from Astra Zeneca and Merck Healthcare KGaA. ES‐H, LRdL, AA‐H, IS, IM‐H, SV, CC, CA, NA, JV, CM‐d‐l‐C, and RR have declared no conflicts of interest.

## Author contributions

AG‐C, ES‐H, LRdL, AR, and MAM‐V conceived and designed the study. AG‐C, ES‐H, LRdL, CA, JV, CM‐d‐l‐C, NA, AR, and MAM‐V conducted the experiments, acquired, and analyzed the data. AA‐H, IS, VC, IM‐H, SV, CC, NR, RR and MP provided clinical samples and collected clinical information. AG‐C, ES‐H, LRdL, AR, and MAM‐V wrote the manuscript. All authors reviewed and/or revised the manuscript.

### Peer review

The peer review history for this article is available at https://www.webofscience.com/api/gateway/wos/peer-review/10.1002/1878-0261.13468.

## Supporting information


**Fig. S1.** Bioanalyzer analysis of RNA samples using the Agilent RNA 6000 Pico assay.
**Fig. S2.** Technical variation of nCounter and dPCR.
**Fig. S3.** Analysis of nCounter counts.
**Fig. S4.** Comparison of the total mRNA counts corresponding to the HK genes in fusion‐positive and ‐negative samples by nCounter.
**Fig. S5.** EV‐RNA analysis.

**Table S1.** Centers participating in the study.
**Table S2.** Cell lines used in the study.
**Table S3.** Results of the stability study in blood samples.
**Table S4.** Primer pool for cDNA preamplification for nCounter.
**Table S5.** Probe panel for nCounter hybridization (Elements Chemistry).
**Table S6.** Primer and probe set for dPCR.
**Table S7.** Minimum fraction of tumor RNA required for detection of fusion and variant splicing transcripts by nCounter, as determined in a dilution bank.
**Table S8.** Results of EV‐RNA testing by nCounter in cell lines.
**Table S9.** Minimum amount of EV‐RNA for fusion detection by nCounter.
**Table S10.** Methodologies used in FFPE tissue‐paired samples for fusion and MetΔex14 splicing detection.
**Table S11.** Concentrations of cfRNA in purified samples, retrotranscription (RT) reactions, and RNA integrity number (RIN), as determined by bioanalyzer.
**Table S12.** Concordance of positive cfRNA samples using nCounter vs. dPCR.
**Table S13.** Methodology used for testing of tumor biopsies with paired EV samples.
**Table S14.** Comparison of techniques used for the detection of fusion and *METΔex14* testing in liquid biopsies.

## Data Availability

All data generated or analyzed during this study are included in this manuscript and its supplementary information files or are available from the authors upon reasonable request.
