# Supplementary material for: Detecting ALK , ROS1, and RET fusions and the METΔex14 splicing variant in liquid biopsies of non‐small‐cell lung cancer patients using RNA‐based techniques
Source: Mol Oncol. 2023 Jun 6;17(9):1884–97. doi: 10.1002/1878-0261.13468 (PMC10483610; doi:10.1002/1878-0261.13468)
Supplement: Supplementary file 1 — Fig. S1. Bioanalyzer analysis of RNA samples using the Agilent RNA 6000 Pico assay. Fig. S2. Technical variation of nCounter and dPCR. Fig. S3. Analysis of nCounter counts. Fig. S4. Comparison of the total mRNA counts corresponding to the HK genes in fusion‐positive and ‐negative samples by nCounter. Fig. S5. EV‐RNA analysis. [file MOL2-17-1884-s001.pptx]

## Slide 1
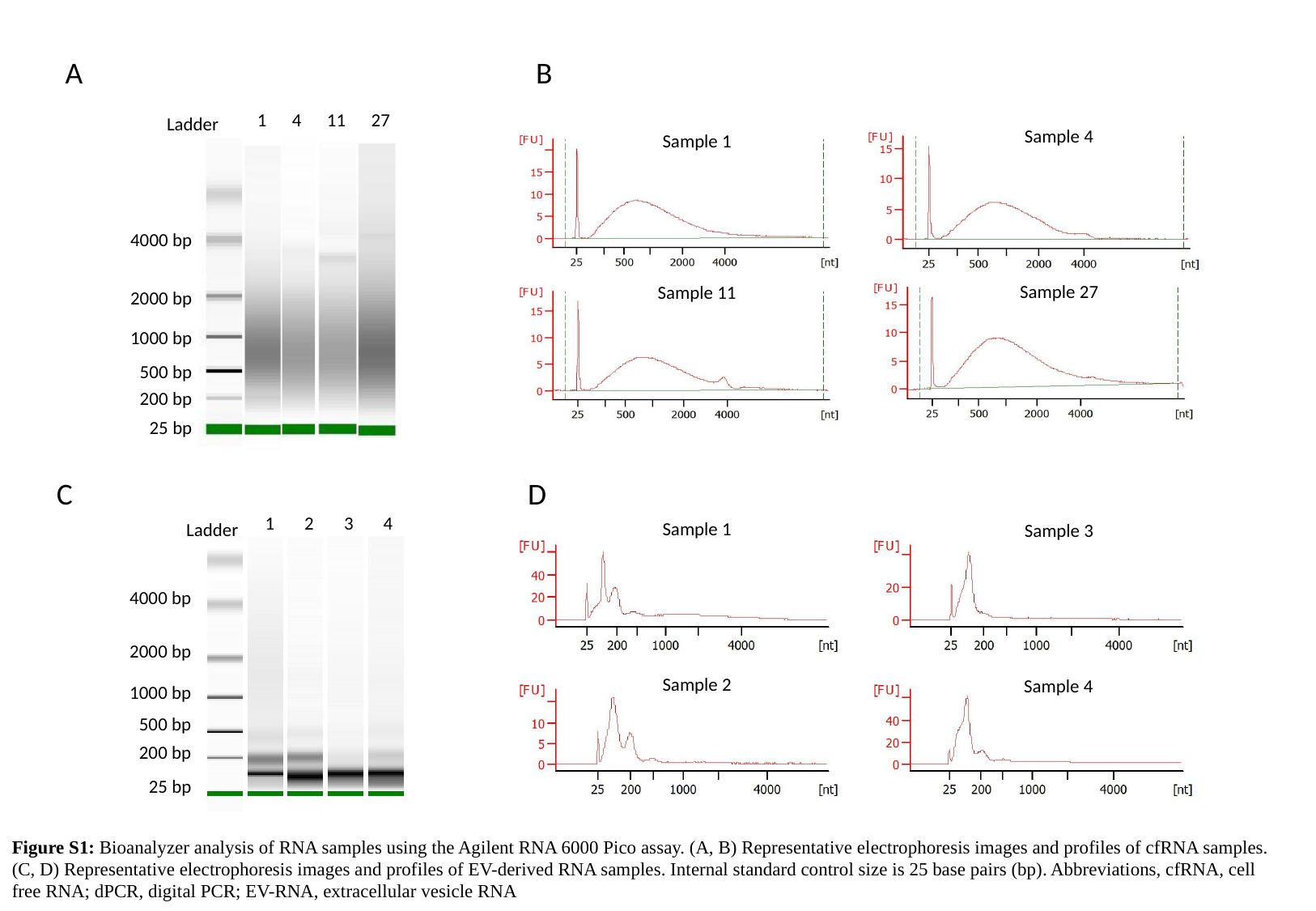

A
B
1 4 11 27
Ladder
4000 bp
2000 bp
1000 bp
500 bp
200 bp
25 bp
Sample 4
Sample 1
Sample 27
Sample 11
C
D
1
2
3
4
Ladder
4000 bp
2000 bp
1000 bp
500 bp
200 bp
25 bp
Sample 1
Sample 3
Sample 2
Sample 4
Figure S1: Bioanalyzer analysis of RNA samples using the Agilent RNA 6000 Pico assay. (A, B) Representative electrophoresis images and profiles of cfRNA samples. (C, D) Representative electrophoresis images and profiles of EV-derived RNA samples. Internal standard control size is 25 base pairs (bp). Abbreviations, cfRNA, cell free RNA; dPCR, digital PCR; EV-RNA, extracellular vesicle RNA

## Slide 2
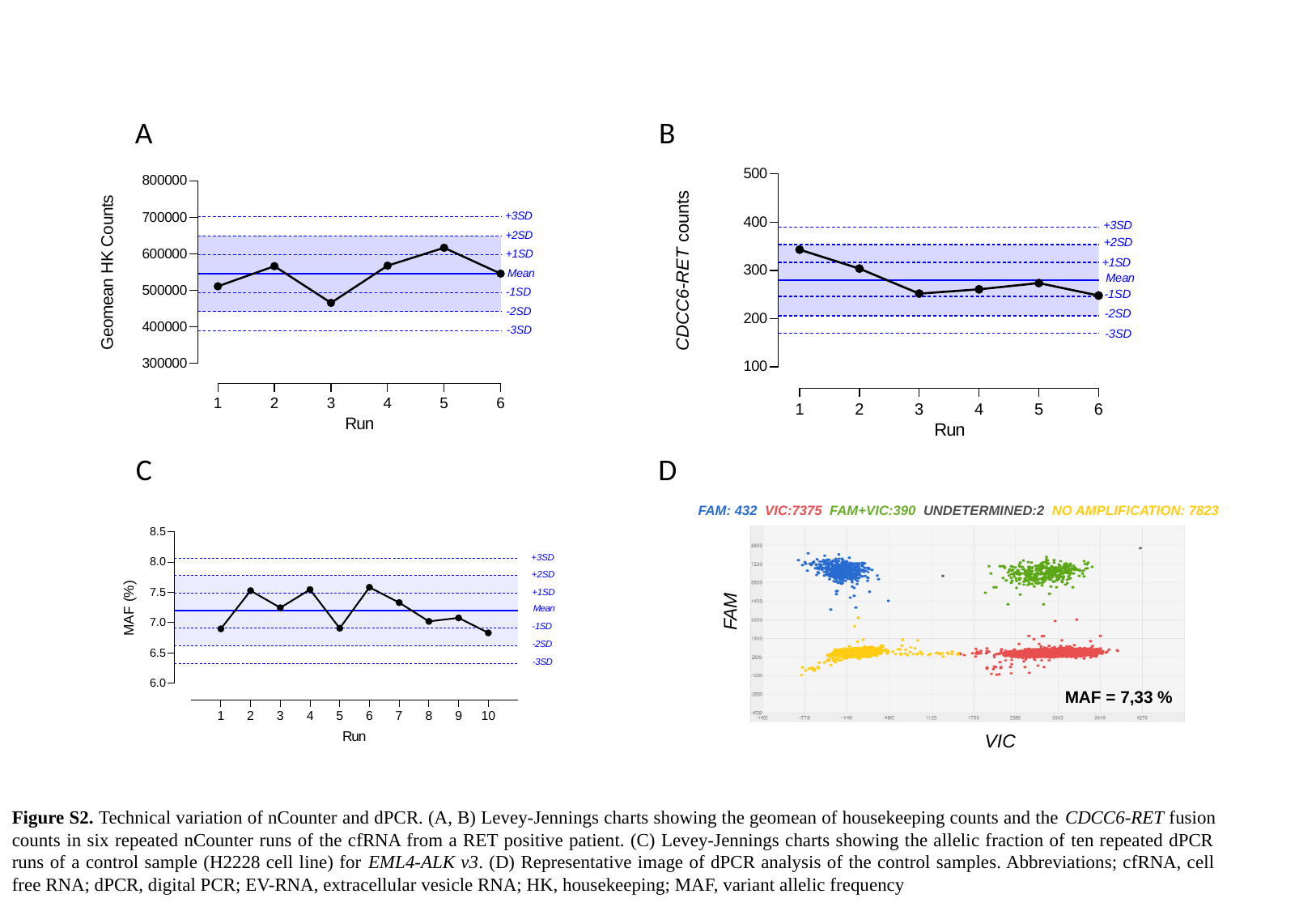

A
B
C
D
FAM: 432 VIC:7375 FAM+VIC:390 UNDETERMINED:2 NO AMPLIFICATION: 7823
FAM
VIC
MAF = 7,33 %
Figure S2. Technical variation of nCounter and dPCR. (A, B) Levey-Jennings charts showing the geomean of housekeeping counts and the CDCC6-RET fusion counts in six repeated nCounter runs of the cfRNA from a RET positive patient. (C) Levey-Jennings charts showing the allelic fraction of ten repeated dPCR runs of a control sample (H2228 cell line) for EML4-ALK v3. (D) Representative image of dPCR analysis of the control samples. Abbreviations; cfRNA, cell free RNA; dPCR, digital PCR; EV-RNA, extracellular vesicle RNA; HK, housekeeping; MAF, variant allelic frequency

## Slide 3
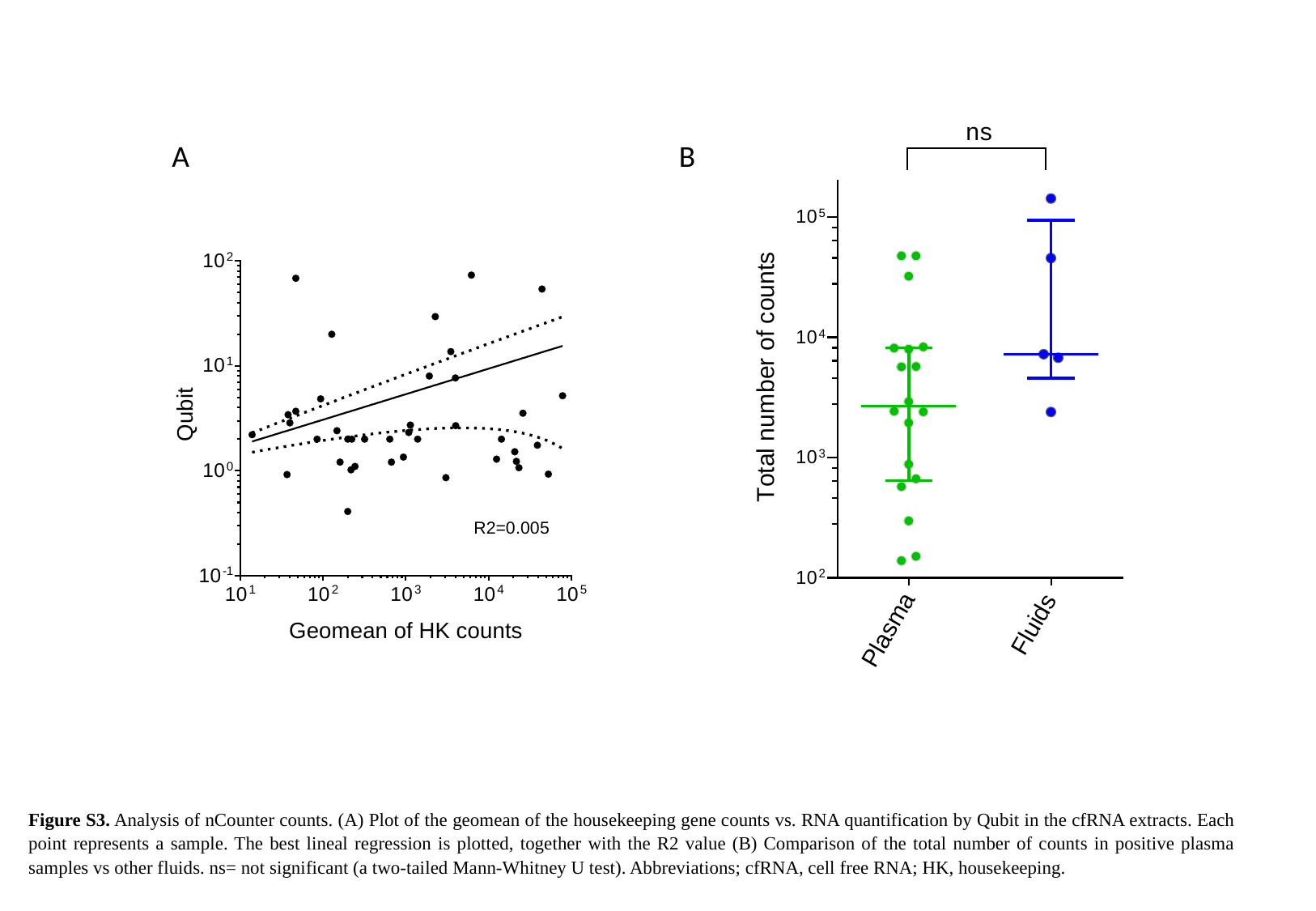

A
B
Figure S3. Analysis of nCounter counts. (A) Plot of the geomean of the housekeeping gene counts vs. RNA quantification by Qubit in the cfRNA extracts. Each point represents a sample. The best lineal regression is plotted, together with the R2 value (B) Comparison of the total number of counts in positive plasma samples vs other fluids. ns= not significant (a two-tailed Mann-Whitney U test). Abbreviations; cfRNA, cell free RNA; HK, housekeeping.

## Slide 4
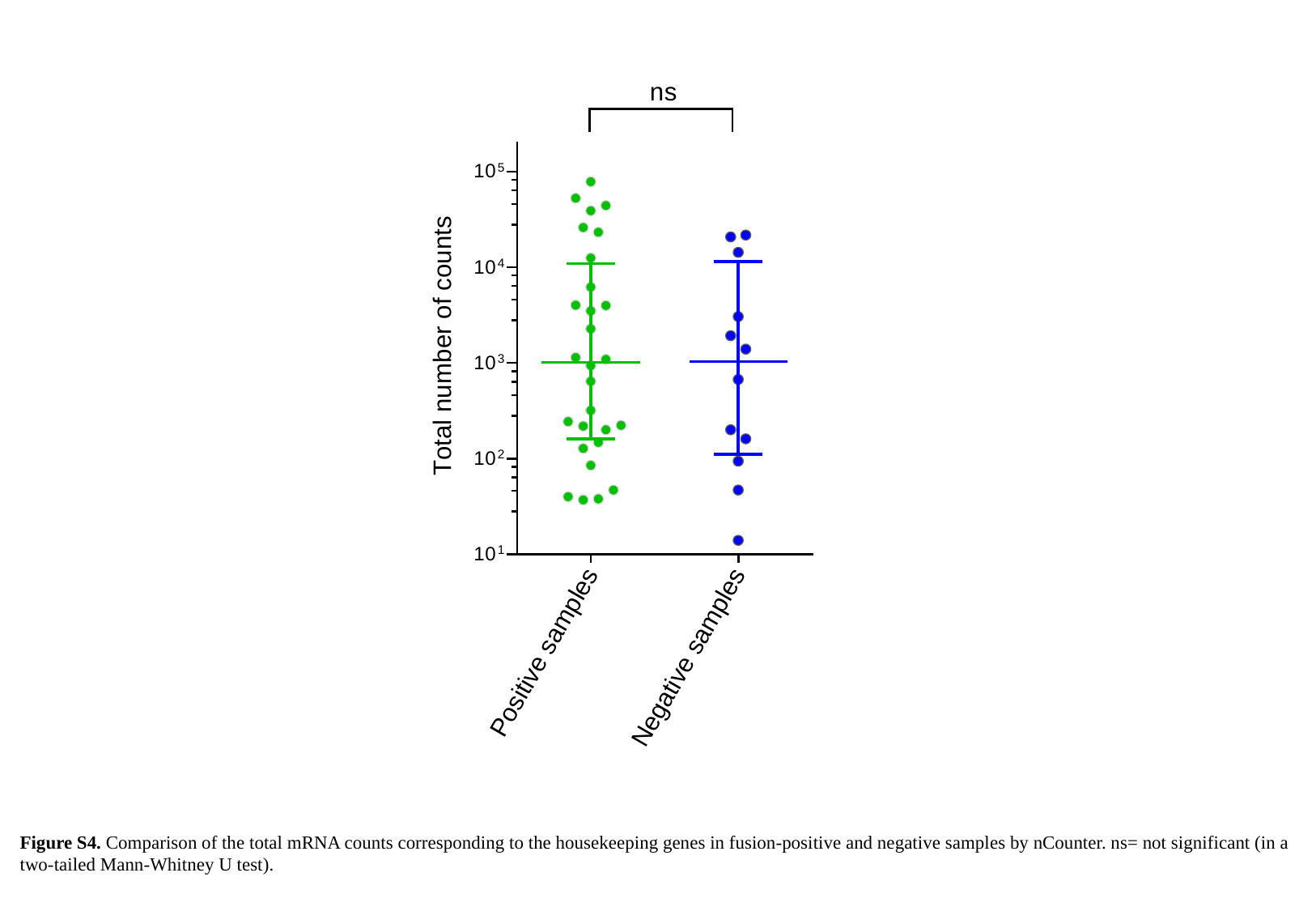

Figure S4. Comparison of the total mRNA counts corresponding to the housekeeping genes in fusion-positive and negative samples by nCounter. ns= not significant (in a two-tailed Mann-Whitney U test).

## Slide 5
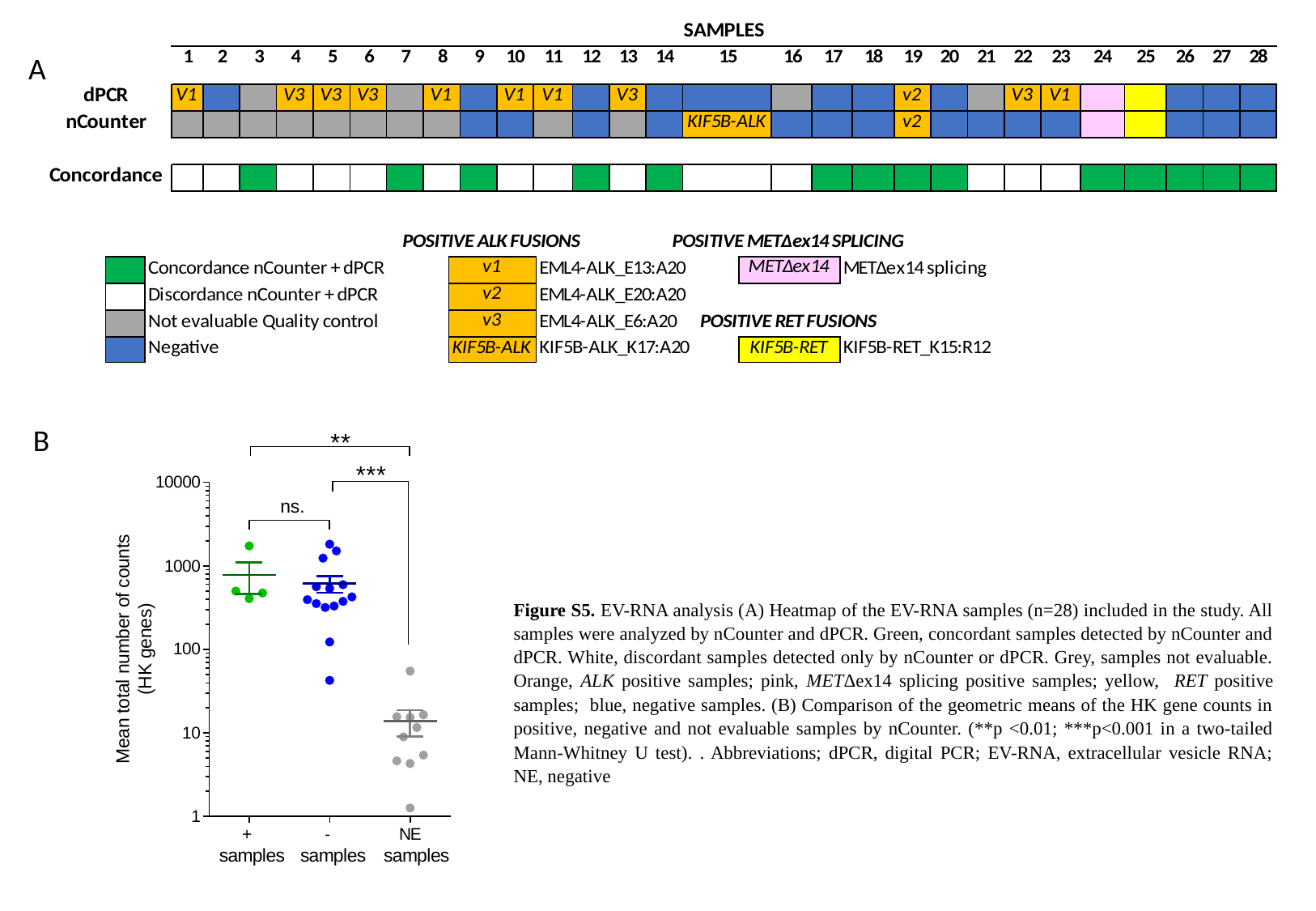

A
B
Figure S5. EV-RNA analysis (A) Heatmap of the EV-RNA samples (n=28) included in the study. All samples were analyzed by nCounter and dPCR. Green, concordant samples detected by nCounter and dPCR. White, discordant samples detected only by nCounter or dPCR. Grey, samples not evaluable. Orange, ALK positive samples; pink, METΔex14 splicing positive samples; yellow, RET positive samples; blue, negative samples. (B) Comparison of the geometric means of the HK gene counts in positive, negative and not evaluable samples by nCounter. (**p <0.01; ***p<0.001 in a two-tailed Mann-Whitney U test). . Abbreviations; dPCR, digital PCR; EV-RNA, extracellular vesicle RNA; NE, negative
