# Supplementary material for: Detecting ALK , ROS1, and RET fusions and the METΔex14 splicing variant in liquid biopsies of non‐small‐cell lung cancer patients using RNA‐based techniques
Source: Mol Oncol. 2023 Jun 6;17(9):1884–97. doi: 10.1002/1878-0261.13468 (PMC10483610; doi:10.1002/1878-0261.13468)
Supplement: Supplementary file 2 — Table S1. Centers participating in the study. Table S2. Cell lines used in the study. Table S3. Results of the stability study in blood samples. Table S4. Primer pool for cDNA preamplification for nCounter. Table S5. Probe panel for nCounter hybridization (Elements Chemistry). Table S6. Primer and probe set for dPCR. Table S7. Minimum fraction of tumor RNA required for detection of fusion and variant splicing transcripts by nCounter, as determined in a dilution bank. Table S8. Results of EV‐RNA testing by nCounter in cell lines. Table S9. Minimum amount of EV‐RNA for fusion detection by nCounter. Table S10. Methodologies used in FFPE tissue‐paired samples for fusion and MetΔex14 splicing detection. Table S11. Concentrations of cfRNA in purified samples, retrotranscription (RT) reactions, and RNA integrity number (RIN), as determined by bioanalyzer. Table S12. Concordance of positive cfRNA samples using nCounter vs. dPCR. Table S13. Methodology used for testing of tumor biopsies with paired EV samples. Table S14. Comparison of techniques used for the detection of fusion and METΔex14 testing in liquid biopsies. [file MOL2-17-1884-s002.docx]

**Supplementary Data**

**Title:** Detection of *ALK, ROS1* and *RET* fusions and *METΔex14* splicing variant in liquid biopsies of non-small cell lung cancer patients using RNA-based techniques

**Table S1.** Centers participating in the study.

| **Hospital** | **City** |
| --- | --- |
| Hospital Universitario Dexeus | Barcelona |
| Hospital Universitario Puerta de Hierro | Majadahonda (Madrid) |
| Hospital General de Catalunya | Sant Cugat del Vallès (Barcelona) |
| Hospital Clínic de Barcelona | Barcelona |
| UOMI Cancer Center, Clínica Mi Tres Torres, Barcelona | Barcelona |
| Centro Médico Teknon | Barcelona |

**Table S2.** Cell lines used in the study. NA, no fusion transcripts or MET∆14 splicing variant. RRID, Research Resource Identifier

| **Cell Line** | **RRID** | **Fusion transcripts / *MET****∆14* **Splicing Variant** | **Histology** |
| --- | --- | --- | --- |
| **NCI-H2228** | CVCL_1543 | *EML4-ALK*_E6:A20 | NSCLC |
| **NCI-H3122** | CVCL_5160 | *EML4-ALK*_E13:A20 | NSCLC |
| **HCC78** | CVCL_2061 | *CD74-ROS1*_C6:R32 | NSCLC |
| **LC-2/ad** | CVCL_1373 | *CCDC6-RET_C1:R11* | NSCLC |
| **Hs 746.T**  **NCI-H23**  **A-549**  **PC-9** | CVCL_0333  CVCL_1547  CVCL_0023  CVCL_B260 | *MET*∆*14* splicing Variant  NA  NA  NA | Gastric  NSCLC  NSCLC  NSCLC |

**Table S3.** Results of the stability study in blood samples. Blood samples were collected simultaneously in K2-EDTA tubes and stored at room temperature for 12 and 24 h. The cut-off positivity value for the EML4-ALK_E6:A20 probe is 150 counts. HK, housekeeping

| **TARGET** | **Initial Counts** | **Counts**  **12 Hours** | **Counts**  **24 Hours** |
| --- | --- | --- | --- |
| **EML4-ALK_E6:A20 positive patient** |  |  |  |
| *EML4-ALK__E13:A20* | 2 | 5 | 3 |
| *EML4-ALK__E18:A20* | 1 | 1 | 1 |
| *EML4-ALK__E6:A20* | 2220 | 2791 | 1273 |
| *EML4-ALK__E2:A20* | 1 | 1 | 1 |
| *EML4-ALK__E20:A20* | 2 | 4 | 1 |
| *KIF5B-ALK__K17:A20* | 1 | 1 | 6 |
| *TFG-ALK__T5:A20* | 1 | 1 | 1 |
| *HK_GAPDH* | 158764 | 93949 | 60870 |
| *HK_MRPL19* | 416108 | 672819 | 878223 |
| *HK_PSMC4* | 1092938 | 1014146 | 789194 |
| HK Geomean | 416407 | 400218 | 348121 |
| **Fusion negative patient** |  |  |  |
| *EML4-ALK__E13:A20* | 1 | 1 | 5 |
| *EML4-ALK__E18:A20* | 1 | 1 | 1 |
| *EML4-ALK__E6:A20* | 2 | 1 | 3 |
| *EML4-ALK__E2:A20* | 1 | 1 | 1 |
| *EML4-ALK__E20:A20* | 1 | 1 | 1 |
| *KIF5B-ALK__K17:A20* | 1 | 3 | 1 |
| *TFG-ALK__T5:A20* | 1 | 5 | 1 |
| *HK_GAPDH* | 68319 | 133923 | 105897 |
| *HK_MRPL19* | 569317 | 189587 | 108609 |
| *HK_PSMC4* | 502024 | 570858 | 706161 |
| HK Geomean | 269282 | 243817 | 201010 |

**Table S4**. Primer pool for cDNA pre-amplification for nCounter.

| **Target** | **Forward** | **Reverse** | **Amplicon Length** |
| --- | --- | --- | --- |
| *GAPDH* | CCGTTGACTCCGACCTTC | CGCTCTCTGCTCCTCCTG | 106 |
| *MRPL19* | GCTCCAAGTCCTCTTCCTGA | TTCTATGTTGGAAGTATTCTTCGTG | 110 |
| *PSMC4* | GGAATTTCTCCATGCCCA | GCCCACGATGGCTGTATT | 103 |
| *EML4-ALK__E13:A20* | GCTCCATCTGCATGGCTT | AGGTGGAGTCATGCTTATATGG | 110 |
| *EML4-ALK__E18:A20* | GGCTCTGCAGCTCCATCT | CCACACAGACGGGAATGA | 101 |
| *EML4-ALK__E2:A20* | GCTCCATCTGCATGGCTT | TTTGAGGCGTCTTGCAATC | 110 |
| *EML4-ALK__E20:A20* | GCTCCATCTGCATGGCTT | CCTTGACTGGTCCCCAGA | 110 |
| *EML4-ALK__E6:A20* | GCTCCATCTGCATGGCTT | CCCAAATTAATACCAAAAGTTACCA | 109 |
| *KIF5B-ALK__K17:A20* | AGCTCCATCTGCATGGCT | TGGAGGAATCTGTCGATGC | 100 |
| *TFG-ALK__T5:A20* | GCTCCATCTGCATGGCTT | CAGGCGTTCAGCCACAG | 108 |
| *CCDC6-RET_C1:R12* | TCCTAGAGTTTTTCCAAGAACCA | GGCACTGCAGGAGGAGAA | 106 |
| *KIF5B-RET_K15:R11* | ACAGCGGCTGCGATCA | GGCATCTTTACTAAAAGACCTTGC | 107 |
| *KIF5B-RET_K16:R12* | TCCTAGAGTTTTTCCAAGAACCA | TGGAAGAAAATGAAAAGGAGTT | 107 |
| *KIF5B-RET_K22:R12* | TCCTAGAGTTTTTCCAAGAACCA | CACAACCTGCGCAAACTCT | 108 |
| *KIF5B-RET_K23:R12* | TCCTAGAGTTTTTCCAAGAACCA | CGCTGCTCAGAAGCAAAA | 127 |
| *KIF5B-RET_K24:R11* | GGAGAAGAGGACAGCGGC | GGAAGCAGTCAGGTCAAAGAA | 109 |
| *KIF5B-RET_K24:R8* | TCTCTTGCTGACTGCACAGG | AGCAGTCAGGTCAAAGAATATGG | 100 |
| *KIF5B_K15-Common* | TCCTAGAGTTTTTCCAAGAACCA | AAAAGACCTTGCAGAAATAGGAA | 106 |
| *KIF5B_K24-Common* | GCTCCATCTGCATGGCTT | TCGCATAAAGGAAGCAGTCA | 107 |
| *SDC4-ROS1_S2:R32* | GCTTTCTCCCACTGTATTGAATTT | CGGGCAGGAATCTGATGA | 120 |
| *SDC4-ROS1_S4:R34* | TGTAACAACCAGAAATATTCCAAC | GGTGTCAATGTCCAGCACTGT | 130 |
| *SLC34A2_S4:ROS1-Common* | TCCCACTGTATTGAATTTTTACTCC | TTCGTGTGCTCCCTGGAT | 115 |
| *SLC34A2-ROS1_S13del2046:R32* | TCCCACTGTATTGAATTTTTACTCC | AAGGCTCCTGAGACCTTTGA | 116 |
| *SLC34A2-ROS1_S4:R32* | TTGTAACAACCAGAAATATTCCAA | TTTTCGTGTGCTCCCTGG | 120 |
| *EZR-ROS1_E10:R34* | TTGTAACAACCAGAAATATTCCAA | GAGGAGTTGATGCTGCGG | 127 |
| *GOPC-ROS1_G4:R36* | TGTCACCCCTTCCTTGG | CCCTGGTGCTAGTTGCAAAG | 100 |
| *GOPC-ROS1_G7:R35* | CCCGAGGGAAGGCAG | TATGGGGCGAGACTAGCTG | 100 |
| *TPM3-ROS1_T8:R35* | CCCCTTCCTTGGCACTTT | CCGTGCTGAGTTTGCTGA | 107 |
| *LRIG3-ROS1 _L16:R35* | CCCCTTCCTTGGCACTTT | GCCACCACCAGTTTGTCA | 108 |
| *CD74-ROS1_C6:R32* | TCCCACTGTATTGAATTTTTACTCC | TGAAATGAGCAGGCACTCC | 120 |
| *MET_e13_14* | TTCGGGCACTTACAAGCC | TTTCCTGTGGCTGAAAAAGAG | 110 |
| *MET_e13_15* | TCAGAGGATACTGCACTTGTCG | CTTGGGTTTTTCCTGTGGC | 100 |

**Table S5**. Probe panel for nCounter hybridization (Elements Chemistry).

| **Assay Type** | **Target Variant** | **Accession Number** | **Target Sequence** |
| --- | --- | --- | --- |
| Fusion | *EML4-ALK__E13:A20* | PFUS_001.1:1 | ATATGGAGCAAAACTACTGTAGAGCCCACACCTGGGAAAGGACCTAAAGTGTACCGCCGGAAGCACCAGGAGCTGCAAGCCATGCAGATGGAGCTGCAG |
| Fusion | *EML4-ALK__E20:A20* | PFUS_002.1:1 | GACAACAAGTATATAATGTCTAACTCGGGAGACTATGAAATATTGTACTTGTACCGCCGGAAGCACCAGGAGCTGCAAGCCATGCAGATGGAGCTGCAG |
| Fusion | *EML4-ALK__E6:A20* | PFUS_003.1:1 | AAAGTTACCAAAACTGCAGACAAGCATAAAGATGTCATCATCAACCAAGTGTACCGCCGGAAGCACCAGGAGCTGCAAGCCATGCAGATGGAGCTGCAG |
| Fusion | *EML4-ALK__E2:A20* | PFUS_006.1:1 | ATCTCTGAAGATCATGTGGCCTCAGTGAAAAAATCAGTCTCAAGTAAAGTGTACCGCCGGAAGCACCAGGAGCTGCAAGCCATGCAGATGGAGCTGCAG |
| Fusion | *EML4-ALK__E18:A20* | PFUS_008.1:1 | ATCCACACAGACGGGAATGAACAGCTCTCTGTGATGCGCTACTCAATAGTGTACCGCCGGAAGCACCAGGAGCTGCAAGCCATGCAGATGGAGCTGCAG |
| Fusion | *TFG-ALK__T5:A20* | PFUS_016.1:1 | CAGCAGCCACCATATACAGGAGCTCAGACTCAAGCAGGTCAGATTGAAGTGTACCGCCGGAAGCACCAGGAGCTGCAAGCCATGCAGATGGAGCTGCAG |
| Fusion | *KIF5B-ALK__K17:A20* | PFUS_031.1:0 | TTGGAGGAATCTGTCGATGCCCTCAGTGAAGAACTAGTCCAGCTTCGAGCACAAGTGTACCGCCGGAAGCACCAGGAGCTGCAAGCCATGCAGATGGAGC |
| Fusion | *EZR-ROS1_E10:R34* | PFUS_032.1:2 | AAGGAGGAGTTGATGCTGCGGCTGCAGGACTATGAGGAGAAGACAAAGAAGGCAGAGAGAGATGATTTTTGGATACCAGAAACAAGTTTCATACTTACTA |
| Fusion | *GOPC-ROS1_G4:R36* | PFUS_023.1:0 | CCCTGGTGCTAGTTGCAAAGACACAAGTGGGGAAATCAAAGTATTACAAGTCTGGCATAGAAGATTAAAGAATCAAAAAAGTGCCAAGGAAGGGGTGACA |
| Fusion | *TPM3-ROS1_T8:R35* | PFUS_035.1:21 | AGTTTGCTGAGAGATCGGTAGCCAAGCTGGAAAAGACAATTGATGACCTGGAAGTCTGGCATAGAAGATTAAAGAATCAAAAAAGTGCCAAGGAAGGGGT |
| Fusion | *LRIG3-ROS1 _L16:R35* | PFUS_027.1:32 | AGTTTGTCACATCTTCAGGTGCTGGATTTTTCTTACCACAACATGACAGTAGTGTCTGGCATAGAAGATTAAAGAATCAAAAAAGTGCCAAGGAAGGGGT |
| Fusion | *GOPC-ROS1_G7:R35* | PFUS_022.1:0 | TATGGGGCGAGACTAGCTGCCAAGTACTTGGATAAGGAACTGGCAGGAAGTACTCTTCCAACCCAAGAGGAGATTGAAAATCTTCCTGCCTTCCCTCGGG |
| Fusion | *SLC34A2-ROS1_S13del2046:R32* | PFUS_034.1:23 | AAGGCTCCTGAGACCTTTGATAACATAACCATTAGCAGAGAGGCTCAGGCTGGAGTCCCAAATAAACCAGGCATTCCCAAATTACTAGAAGGGAGTAAA |
| Fusion | *CD74-ROS1_C6:R32* | PFUS_030.1:23 | AATGAGCAGGCACTCCTTGGAGCAAAAGCCCACTGACGCTCCACCGAAAGCTGGAGTCCCAAATAAACCAGGCATTCCCAAATTACTAGAAGGGAGTAAA |
| Fusion | *SDC4-ROS1_S2:R32* | PFUS_024.1:35 | GCCCGGGCAGGAATCTGATGACTTTGAGCTGTCTGGCTCTGGAGATCTGGCTGGAGTCCCAAATAAACCAGGCATTCCCAAATTACTAGAAGGGAGTAAA |
| Fusion | *SDC4-ROS1_S4:R34* | PFUS_033.1:7 | GGTGTCAATGTCCAGCACTGTGCAGGGCAGCAACATCTTTGAGAGAACGGAGGTCCTGGCAGATGATTTTTGGATACCAGAAACAAGTTTCATACTTACT |
| Fusion | *SLC34A2_S4:ROS1-Common* | PFUS_020.1:5 | GTGTGCTCCCTGGATATTCTTAGTAGCGCCTTCCAGCTGGTTGGAGCTGGAGTCCCAAATAAACCAGGCATTCCCAAATTACTAGAAGGGAGTAA |
| Fusion | *KIF5B-RET_K16:R12* | PFUS_025.1:7 | AAGAAAATGAAAAGGAGTTAGCAGCATGTCAGCTTCGTATCTCTCAAGAGGATCCAAAGTGGGAATTCCCTCGGAAGAACTTGGTTCTTGGAAAAACTCT |
| Fusion | *KIF5B-RET_K22:R12* | PFUS_026.1:12 | ACCTGCGCAAACTCTTTGTTCAGGACCTGGCTACAAGAGTTAAAAAGGAGGATCCAAAGTGGGAATTCCCTCGGAAGAACTTGGTTCTTGGAAAAACTCT |
| Fusion | *KIF5B-RET_K23:R12* | PFUS_029.1:51 | CCTTTCTTGAAAATAATCTTGAACAGCTCACTAAAGTGCACAAACAGGAGGATCCAAAGTGGGAATTCCCTCGGAAGAACTTGGTTCTTGGAAAAACTCT |
| Fusion | *CCDC6-RET_C1:R12* | PFUS_039.1:10 | GGAGGAGAACCGCGACCTGCGCAAAGCCAGCGTGACCATCGAGGATCCAAAGTGGGAATTCCCTCGGAAGAACTTGGTTCTTGGAAAAAC |
| Fusion | *KIF5B_K24-Common* | PFUS_013.1:1 | GCAGTCAGGTCAAAGAATATGGCCAGAAGAGGGCATTCTGCACAGATTGTGTACCGCCGGAAGCACCAGGAGCTGCAAGCCATGCAGATGGAGCTGCAG |
| Fusion | *KIF5B_K15-Common* | PFUS_028.1:40 | AAGACCTTGCAGAAATAGGAATTGCTGTGGGAAATAATGATGTAAAGGAGGATCCAAAGTGGGAATTCCCTCGGAAGAACTTGGTTCTTGGAAAAACTCT |
| *METΔex14* | *MET* | RCC_AS01_065.1:27_T053 | TCCTGTGGCTGAAAAAGAGAAAGCAAATTAAAGATCAGTTTCCTAATTCATCTCAGAACGGTTCATGCCGACAAGTGCAGTATCCTCTGACAG |
| *MET wt* | *MET* | RCC_AS01_066.2:1_T052 | CCTGTGGCTGAAAAAGAGAAAGCAAATTAAAGATCTGGGCAGTGAATTAGTTCGCTACGATGCAAGAGTACACACTCCTCATTTGGATAGGC |
| Endogenous | *GAPDH* | NM_002046.3:35_T001 | TCCTCCTGTTCGACAGTCAGCCGCATCTTCTTTTGCGTCGCCAGCCGAGCCACATCGCTCAGACACCATGGGGAAGGTGAAGGTCGGAGTCAACGGATTT |
| Endogenous | *MRPL19* | NM_014763.3:364_T003 | GGAAGTATTCTTCGTGTTACTACAGCTGACCCATATGCCAGTGGAAAAATCAGCCAGTTTCTGGGGATTTGCATTCAGAGATCAGGAAGAGGACTTGGAG |
| Endogenous | *PSMC4* | NM_006503.2:250_T004 | TTTCTCCATGCCCAGGAGGAGGTGAAGCGAATCCAAAGCATCCCGCTGGTCATCGGACAATTTCTGGAGGCTGTGGATCAGAATACAGCCATCGTGGGCT |

**Table S6**. Primer and probe set for digital PCR (dPCR).

| **Target type** | **Genomic alteration/Gene** | **dPCR TaqMan® Assays** | **Dye** | **Amplicon Length** |
| --- | --- | --- | --- | --- |
| Fusion | *CCDC6-RET_C1:R12* | Hs04396844_ft | FAM-MGB | 89 |
| Fusion | *CD74-ROS1_C6:R32* | Hs04396895_ft | FAM-MGB | 110 |
| Fusion | *EML4-ALK_E13:A20* | Hs03654556_ft | FAM-MGB | 79 |
| Fusion | *EML4-ALK_E18:A20* | Hs03654559_ft | FAM-MGB | 80 |
| Fusion | *EML4-ALK_E20:A20* | Hs03654557_ft | FAM-MGB | 86 |
| Fusion | *EML4-ALK_E6a/b:A20* | Hs03654558_ft | FAM-MGB | 85 |
| Fusion | *KIF5B-RET_K15:R12* | Hs04396863_ft | FAM-MGB | 102 |
| Fusion | *KIF5B-ALK_K17:A20* | Hs04397082_ft | FAM-MGB | 86 |
| *MET wt* | *MET wild-type* | CCCGAAGTGTAAGCCCAA | VIC-MGB | 247 |
| *METΔex14* | *MET exon skipping 14* | CAAATTAAAGATCAGTTTC | FAM-MGB | 106 |
| Endogenous gene | *PUM1* | Hs00472881_m1 | VIC-MGB | 77 |
| Fusion | *SCD4-ROS1_S2:R32* | Hs04396929_ft | FAM-MGB | 117 |
| Fusion | *SCL34A2-ROS1_S4:A32* | Hs04396941_ft | FAM-MGB | 112 |

**Table S7**. Minimum fraction of tumor RNA required for detection of fusion and variant splicing transcripts by nCounter, as determined in a dilution bank. The starting material for the bank was a mixture of RNA from five positive cell lines (10 µL each) with circulating-free RNA (cfRNA) from the plasma of a healthy individual (50 µL). The concentrations of the cell line and plasma samples in the starting material were all 100 ng/µL. Consequently, the dilution factor of every cell line RNA was 1/10 (1/5 x 1/2). The mixture was then serially diluted into the same cfRNA preparation from the healthy individual.

|  |  |  | **Serial dilution** | | | |
| --- | --- | --- | --- | --- | --- | --- |
|  |  | Dilution factor of starting material | 1 | 1/10 | 1/100 | 1/1000 |
|  |  | Dilution factor of each cell line RNA | 1/10 | 1/100 | 1/1000 | 1/10000 |
| **Cell line** | **Target** | Fraction of each cell line RNA | 10% | 1% | 0.1% | 0.01% |
| **H3122** | *EML4-ALK_ E13:A20* | Count value | 36321 | 66056 | 9339 | 36 |
|  |  | Results | Detected | Detected | Detected | undetected |
| **H2228** | *EML4-ALK_ E16:A20* | Count value | 207454 | 18818 | 6674 | 43 |
|  |  | Results | Detected | Detected | Detected | undetected |
| **HCC78** | *SLC34A2_S4:ROS1* | Count value | 58432 | 126934 | 37557 | 2647 |
|  |  | Results | Detected | Detected | Detected | Detected |
| **LC2ad** | *CCDC6-RET* | Count value | 3500 | 1417 | 40 | 2 |
|  |  | Results | Detected | Detected | undetected | undetected |
| **Hs746T** | *METΔex14* | Ratio count value | 1.09 | 0.97 | 0.71 | 0.50 |
|  |  | Results | Detected | Detected | Detected | Detected |

**Table S8**. Results of extracellular vesicle RNA (EV-RNA) testing by nCounter in cell lines. Five cell lines harboring *ALK*, *ROS1* and *RET* rearrangements and *MET∆14* splicing variant and two cell lines without fusions or splicing variant described were used.

| **Cell lines** | **Target** | **Count Value** | **Result** |
| --- | --- | --- | --- |
| **H3122** | *EML4-ALK__E13:A20* | 1652 | Detected |
| **H2228** | *EML4-ALK__E6:A20* | 670 | Detected |
| **HCC78** | *SLC34A2_S4:ROS1* | 4150 | Detected |
| **LC2-ad** | *CCDC6-RET_C1:R12* | 5404 | Detected |
| **Hs746T** | *METΔex14* | ratio:1.00 | Detected |
| **H23** | *EML4-ALK__E13:A20* | 21 | Undeteted |
|  | *EML4-ALK__E6:A20* | 14 | Undeteted |
|  | *SLC34A2_S4:ROS1* | 10 | Undeteted |
|  | *CCDC6-RET_C1:R12* | 5 | Undeteted |
|  | *METΔex14* | ratio: 0.45 | Undeteted |
| **A549** | *EML4-ALK__E13:A20* | 10 | Undeteted |
|  | *EML4-ALK__E6:A20* | 6 | Undeteted |
|  | *SLC34A2_S4:ROS1* | 6 | Undeteted |
|  | *CCDC6-RET_C1:R12* | 7 | Undeteted |
|  | *METΔex14* | ratio:0.43 | Undeteted |

**Table S9**. Minimum amount of extracellular vesicle RNA (EV-RNA) for fusion detection by nCounter, as determined using the HCC78 cell line (*ROS1* positive) spiked with RNA from the PC9 cell line (*EGFR*-mutated, wild type for fusions).

| **Cell line** |  | **Serial dilution** | | | | |
| --- | --- | --- | --- | --- | --- | --- |
| **HCC78** | Concentration | 50ng | 5ng | 500pg | 50pg | 5pg |
|  | Counts Value | 88454 | 49574 | 102475 | 39864 | 10146 |
|  | Result | Detected | Detected | Detected | Detected | Detected |

**Table S10**. Methodologies used in FFPE tissue paired samples for fusion and *MetΔex14* splicing detection. NGS was performed using the QIAseq RNA Fusion XP Panel (Qiagen, Hilden, Germany) and run in the miSeq platform (Illumina, Santa Clara, CA). nCounter, FISH and IHC were performed as previously described (1, 2)

| **cfRNA samples** | **TARGET** | **Methodology of tissue testing** |
| --- | --- | --- |
| **Sample 1** | *EML4-ALK* v1 (E18:A20) | nCounter/FISH/IHC |
| **Sample 2** | *EML4-ALK* v1 (E13:A20) | nCounter/IHC |
| **Sample 3** | *EML4-ALK* v1 (E6:A20) | nCounter/FISH |
| **Sample 4** | *EML4-ALK* v1 (E13:A20) | nCounter/FISH |
| **Sample 5** | *METΔex14* | nCounter/NGS |
| **Sample 6** | *METΔex14* | nCounter/NGS |
| **Sample 7** | *METΔex14* | nCounter/NGS |
| **Sample 8** | *CD74-ROS1-C6:R32* | nCounter/FISH |
| **Sample 9** | *CD74-ROS1-C6:R32* | nCounter/FISH |
| **Sample 10** | *CD74-ROS1-C6:R32* | nCounter/FISH |
| **Sample 11** | *EML4-ALK* v1 (E6:A20) | nCounter/IHC |
| **Sample 12** | *EML4-ALK* v1 (E6:A20) | nCounter |
| **Sample 13** | *ALK* | FISH |
| **Sample 14** | *ALK* | FISH |
| **Sample 15** | *ALK* | FISH |
| **Sample 16** | *ALK*) | FISH |
| **Sample 17** | *METΔex14* | nCounter/NGS |
| **Sample 18** | *METΔex14* | nCounter/NGS |
| **Sample 19** | *ALK* | FISH |
| **Sample 20** | *KIF5B_RET_K15:R12* | nCounter/FISH |
| **Sample 21** | *KIF5B_RET_K15:R12* | nCounter/FISH |
| **Sample 22** | *KIF5B_RET_K15:R12* | nCounter/FISH |
| **Sample 23** | *CCDC6-RET_C1:R12* | nCounter/FISH |
| **Sample 24** | *CCDC6-RET_C1:R12* | nCounter/FISH |
| **Sample 25** | *ROS1* | FISH |
| **Sample 26** | *ROS1* | FISH |
| **Sample 27** | *ROS1* | FISH |
| **Sample 28** | *ALK* | FISH |
| **Sample 29** | *EML4-ALK* v1 (E13:A20) | nCounter/IHC |
| **Sample 30** | *RET* | FISH |
| **Sample 31** | *RET* | FISH |
| **Sample 32** | *KIF5B_RET_K15:R12* | nCounter/FISH |
| **Sample 33** | *ROS1* | nCounter |
| **Sample 34** | *RET* | FISH |
| **Sample 35** | *KIF5B_RET_K15:R12* | nCounter/FISH |
| **Sample 36** | *KIF5B_RET_K15:R12* | nCounter/FISH |
| **Sample 37** | *KIF5B_RET_K15:R12* | nCounter/FISH |
| **Sample 38** | *ALK* | FISH |
| **Sample 39** | *ROS1* | FISH |
| **Sample 40** | *CCDC6-RET_C1:R12* | nCounter/FISH |

**Table S11**. Concentrations of cell-free RNA (cfRNA) in purified samples, retrotranscription reactions and RNA integrity number, as determined by bioanalyzer. HK, housekeeping; NA, not analyzed; RIN, RNA integrity number; RT, retrotranscription

|  | **Type of sample** | **Geomean of HK genes** | **ng/µL cfRNA in purified samples** | **ng/µL of input cfRNA in the RT** | **RIN of cfRNA** |
| --- | --- | --- | --- | --- | --- |
| **Sample 1** | Plasma | 85 | 2.5 | 2.5 | 2.3 |
| **Sample 2** | Plasma | 47 | 68.4 | 2.5 | na |
| **Sample 3** | Plasma | 3975 | 7.6 | 2.5 | NA |
| **Sample 4** | Plasma | 12491 | 1.3 | 1.3 | 2.2 |
| **Sample 5** | Plasma | 38 | 3.4 | 0.8 | NA |
| **Sample 6** | Pleural effusion | 6205 | 73.4 | 2.5 | NA |
| **Sample 7** | Plasma | 148 | 2.4 | 2.4 | 2.3 |
| **Sample 8** | Pleural effusion | 3503 | 13.7 | 2.5 | NA |
| **Sample 9** | Plasma | 25967 | 3.5 | 2.5 | NA |
| **Sample 10** | Plasma | 40 | 2.9 | 1.1 | NA |
| **Sample 11** | Pleural effusion | 128 | 20 | 2.5 | 2.5 |
| **Sample 12** | Plasma | 161 | 1.2 | 1.2 | NA |
| **Sample 13** | Cerebrospinal fluid | 44136 | 54 | 2.5 | NA |
| **Sample 14** | Cerebrospinal fluid | 2272 | 29.5 | 2.5 | NA |
| **Sample 15** | Cerebrospinal fluid | 4018 | 2.7 | 2.5 | 2.3 |
| **Sample 16** | Plasma | 38880 | 1.7 | 1.7 | NA |
| **Sample 17** | Plasma | 244 | 1.1 | 1.1 | 2.3 |
| **Sample 18** | Pleural effusion | 1088 | 2.3 | 2.3 | NA |
| **Sample 19** | Plasma | 52699 | 0.9 | 0.9 | NA |
| **Sample 20** | Plasma | 319 | 1.2 | 1.2 | NA |
| **Sample 21** | Plasma | 200 | 0.4 | 0.4 | NA |
| **Sample 22** | Plasma | 23249 | 1.0 | 1.0 | NA |
| **Sample 23** | Plasma | 37 | 0.9 | 0.9 | NA |
| **Sample 24** | Plasma | 219 | 1.0 | 1.0 | NA |
| **Sample 25** | Plasma | 78208 | 5.1 | 2.5 | 2.3 |
| **Sample 26** | Plasma | 643 | 2.5 | 2.5 | NA |
| **Sample 27** | Plasma | 1140 | 2.7 | 2.5 | 2.3 |
| **Sample 28** | Plasma | 200 | 2 | 2.0 | NA |
| **Sample 29** | Plasma | 3056 | 0.8 | 0.8 | NA |
| **Sample 30** | Plasma | 14287 | 2 | 2 | NA |
| **Sample 31** | Plasma | 1395 | 2.5 | 2.5 | 2.3 |
| **Sample 32** | Plasma | 671 | 1.2 | 1.2 | 2.3 |
| **Sample 33** | Plasma | 1926 | 8 | 8 | NA |
| **Sample 34** | Pleural effusion | 94 | 4.8 | 2.5 | NA |
| **Sample 35** | Plasma | 47 | 37 | 2.5 | NA |
| **Sample 36** | Plasma | 14 | 2.2 | 2.2 | NA |
| **Sample 37** | Plasma | 223 | 2 | 2 | NA |
| **Sample 38** | Plasma | 20749 | 1.5 | 1.5 | NA |
| **Sample 39** | Plasma | 21696 | 1.2 | 1.2 | NA |
| **Sample 40** | Plasma | 939 | 1.3 | 1.3 | NA |

**Table S12**. Concordance of positive cell-free RNA (cfRNA) samples using nCounter vs. digital PCR (dPCR)

| **Genes** | ***ALK/ROS1/RET/MET****Δ****ex14*** | ***ALK*** |
| --- | --- | --- |
| No. concordant samples | 23 | 10 |
| No. discordant samples | 17 | 4 |
| Concordance | 57.5% | 71.1% |

**Table S13**: Methodology used for testing of tumor biopsies with paired EV samples. Fusions and *METΔex14* were identified by NGS, FISH, IHC and/or qPCR on FFPE tumor tissue samples. NGS was performed using the Oncomine™ Focus Assay (Thermofisher Scientific) to determine the specific alterations. Briefly, RNA from 5 µm FFPE slides was isolated using truXTRAC® FFPE total Nucleic Acid (Covaris) according to the manufacturer's recommendations, and the obtained RNA was subsequently reverse-transcribed into cDNA by PrimeScript RT Reagent Kit (TaKaRa) and used for NGS analysis.

| **Sample ID** | **Methodology of tissue testing** | **Fusion detected (tissue)** |
| --- | --- | --- |
| Sample 1 | NGS/IHC | *EML4-ALK* v1 (E13:A20) |
| Sample 2 | NGS/IHC | *EML4-ALK* v1 (E13:A20) |
| Sample 3 | NGS/IHC | *EML4-ALK* v1 (E13:A20) |
| Sample 4 | NGS/IHC | *EML4-ALK* v3 (E6:A20) |
| Sample 5 | NGS/IHC | *EML4-ALK* v3 (E6:A20) |
| Sample 6 | NGS/IHC | *EML4-ALK* v3 (E6:A20) |
| Sample 7 | NGS/IHC | *EML4-ALK* v1 (E13:A20) |
| Sample 8 | FISH | - |
| Sample 9 | FISH | - |
| Sample 10 | FISH | - |
| Sample 11 | FISH | - |
| Sample 12 | FISH | - |
| Sample 13 | FISH | - |
| Sample 14 | NGS/IHC | *EML4-ALK* v1 (E13:A20) |
| Sample 15 | NGS/IHC | *KIF5B-ALK* |
| Sample 16 | NGS/IHC | *EML4-ALK* v1 (E13:A20) |
| Sample 17 | NGS/IHC | *EML4-ALK* v1 (E13:A20) |
| Sample 18 | NGS/IHC | *EML4-ALK* v1 (E13:A20) |
| Sample 19 | IHC | - |
| Sample 20 | FISH | - |
| Sample 21 | FISH | - |
| Sample 22 | FISH | - |
| Sample 23 | FISH | - |
| Sample 24 | qPCR | *METex14* |
| Sample 25 | FISH | - |
| Sample 26 | FISH | - |
| Sample 27 | FISH | - |
| Sample 28 | FISH | - |

**Table S14**. Comparison of techniques used for the detection of fusion and *METΔex14* testing in liquid biopsies

| **Characteristics** | **NanoString Technologies** | **FoundationOne Medicine** | **Resolution Bioscience** | **Guardant Health** | **ThermoFisher Scientific** | **ThermoFisher Scientific** | **NeoGenomics** | **Roche** | **RT- PCR** |
| --- | --- | --- | --- | --- | --- | --- | --- | --- | --- |
| **Assay** | Custom panel | FoundationOne  Liquid CDx | Resolution ctDx Lung | Guardant360 CDx assay | Oncomine Lung cfTNA assay | Ion Ampliseq RNA Fusion Lung Cancer panel | InVisionFirst-Lung | Cobas 4800 system | PCR Taqman assays (Life Technologies) |
| **Input requirements** | 1 tube of whole blood | two 10-mL tubes of whole blood | two 10-mL tubes of whole blood | two 10-mL tubes of whole blood | 1 tube of whole blood | 1mL | two 10-mL tubes of whole blood | 8 mL of Whole blood | 6 mL of whole blood |
| **Type of Sample** | cfRNA | cfDNA | cfDNA | cfDNA | cfDNA/cfRNA | cfRNA | cfDNA | cfRNA | cfRNA |
| **Simultaneous detection of SNV/CNV/fusions** | Yes | Yes | Yes | Yes | Yes | No | Yes | No | No |
| **Test turnaround time** | 2 days | 15 days | 9 days | 7 days | 4 days | not reported | 5 days | 1 day | 1 day |
| **Reportable range** | 0.1 - 0.01% | 0.125% - 0.5% | 1.4%-8% | 0.05 - 0.20% | 0.02%-2.2% * | not reported | 0.06% | not reported | not reported |
| **Sensitivity** | 70%  (95% CI= 54.6 - 82) | 92.9% for *ALK*  *RET and ROS1 not reported*  *68.4% to 100%* | 81% for fusions | *Datasheet:*  *ALK, RET, ROS1* (n=37) 83.0%-100%  *Published:*18-44% | 8% - 100% * | *ALK* (n=9) 64% ** | 67% for *ALK* and *ROS1* | 33.33% (95% CI: 17.3–52.8%) | (n=32) 21.0% in plasma (n= 67) 65.0% in platelets |
| **Specificity** | 100%  (95% CI= 97.8 - 100) | 100% for *ALK*  *RET and ROS1 not reported* | Not reported | *Fusions* (n=37) 100% *METΔex14* (n=3) 100% | 100% * | 100% ** | 100% | 100% (95% CI: 85.8–100%) | 100% plasma and platelets |
| **Genes in the panel** | *ALK, RET, ROS1* and *METΔex14* | *ALK, RET, ROS1* and *METΔex14* | *ALK, RET, ROS1* and *METΔex14* | *ALK, RET, ROS1* and *METΔex14* | *ALK, RET, ROS1* and *METΔex14* | *ALK* | *ALK, RET, ROS1* and *METΔex14* | *ALK* and *RET* | *ALK* |
| **Study (author, year, reference)** | **This manuscript** | (3) | (4) | (5)  (6, 7) | (8) | (9) | (10) | (11) | (12) |

*No clinical research samples were available to verify the analytical sensitivity.

** Data from extracellular vesicle RNA (EV-RNA).

SUPPLEMENTARY REFERENCES

1. Aguado C, Teixido C, Roman R, Reyes R, Gimenez-Capitan A, Marin E, Cabrera C, et al. Multiplex RNA-based detection of clinically relevant MET alterations in advanced non-small cell lung cancer. Mol Oncol 2021 Feb;15 2:350-63 as doi: 10.1002/1878-0261.12861.

2. Reguart N, Teixido C, Gimenez-Capitan A, Pare L, Galvan P, Viteri S, Rodriguez S, et al. Identification of ALK, ROS1, and RET Fusions by a Multiplexed mRNA-Based Assay in Formalin-Fixed, Paraffin-Embedded Samples from Advanced Non-Small-Cell Lung Cancer Patients. Clin Chem 2017 Mar;63 3:751-60 as doi: 10.1373/clinchem.2016.265314.

3. Milbury CA, Creeden J, Yip WK, Smith DL, Pattani V, Maxwell K, Sawchyn B, et al. Clinical and analytical validation of FoundationOne(R)CDx, a comprehensive genomic profiling assay for solid tumors. PLoS One 2022;17 3:e0264138 as doi: 10.1371/journal.pone.0264138.

4. Supplee JG, Milan MSD, Lim LP, Potts KT, Sholl LM, Oxnard GR, Paweletz CP. Sensitivity of next-generation sequencing assays detecting oncogenic fusions in plasma cell-free DNA. Lung Cancer 2019 Aug;134:96-9 as doi: 10.1016/j.lungcan.2019.06.004.

5. <https://www.guardanthealth.es/?gclid=EAIaIQobChMInvGoiNWC-gIVy-7tCh074AFVEAAYASAAEgLrEfD_BwE>.

6. Berchuck JE, Facchinetti F, DiToro DF, Baiev I, Majeed U, Reyes S, Chen C, et al. The clinical landscape of cell-free DNA alterations in 1671 patients with advanced biliary tract cancer. Ann Oncol 2022 Dec;33 12:1269-83 as doi: 10.1016/j.annonc.2022.09.150.

7. Hasegawa N, Kohsaka S, Kurokawa K, Shinno Y, Takeda Nakamura I, Ueno T, Kojima S, et al. Highly sensitive fusion detection using plasma cell-free RNA in non-small-cell lung cancers. Cancer Sci 2021 Oct;112 10:4393-403 as doi: 10.1111/cas.15084.

8. R C. Characterization of Genetic Mutation Spectra and Identification of Gene Amplification and Fusion Variants in Cell-Free NucleiAcid from Cultured Cancer Cell Media and Liquid Biopsy Specimens Using Oncomine™ Pan-Cancer Cell-Free Assay. 2019.

9. Reclusa P, Laes JF, Malapelle U, Valentino A, Rocco D, Gil-Bazo I, Rolfo C. EML4-ALK translocation identification in RNA exosomal cargo (ExoALK) in NSCLC patients: a novel role for liquid biopsy. Transl Cancer Res 2019 Jan;8 Suppl 1:S76-S8 as doi: 10.21037/tcr.2018.11.35.

10. Mezquita L, Swalduz A, Jovelet C, Ortiz-Cuaran S, Howarth K, Planchard D, Avrillon V, et al. Clinical Relevance of an Amplicon-Based Liquid Biopsy for Detecting ALK and ROS1 Fusion and Resistance Mutations in Patients With Non-Small-Cell Lung Cancer. JCO Precis Oncol 2020;4 as doi: 10.1200/PO.19.00281.

11. Heeke S, Benzaquen J, Vallee A, Allegra M, Mazieres J, Fayada J, Rajamani J, et al. Detection of ALK fusion transcripts in plasma of non-small cell lung cancer patients using a novel RT-PCR based assay. Ann Transl Med 2021 Jun;9 11:922 as doi: 10.21037/atm-20-7900.

12. Nilsson RJ, Karachaliou N, Gimenez-Capitan A, Schellen P, Teixido C, Tannous J, et al. Rearranged EML4-ALK fusion transcripts sequester in circulating blood platelets and enable blood-based crizotinib response monitoring in non-small-cell lung cancer. Oncotarget 2016 Jan 5;7 1:1066-75 as doi: 10.18632/oncotarget.6279.
